# The Arabidopsis R‐SNARE VAMP714 is essential for polarisation of PIN proteins and auxin responses

**DOI:** 10.1111/nph.17205

**Published:** 2021-02-10

**Authors:** Xiaoyan Gu, Kumari Fonseka, Julien Agneessens, Stuart A. Casson, Andrei Smertenko, Guangqin Guo, Jennifer F. Topping, Patrick J. Hussey, Keith Lindsey

**Affiliations:** ^1^ Department of Biosciences Durham University South Road Durham DH1 3LE UK; ^2^ Ministry of Education Key Laboratory of Cell Activities and Stress Adaptations School of Life Sciences Lanzhou University Lanzhou 730000 China

**Keywords:** *Arabidopsis thaliana*, auxin transport, PIN proteins, R‐SNARE, VAMP714

## Abstract

- The plant hormone auxin and its directional intercellular transport play a major role in diverse aspects of plant growth and development. The establishment of auxin gradients requires the asymmetric distribution of members of the auxin efflux carrier PIN‐FORMED (PIN) protein family to the plasma membrane. An endocytic pathway regulates the recycling of PIN proteins between the plasma membrane and endosomes, providing a mechanism for dynamic localisation.
- *N*‐Ethylmaleimide‐sensitive factor adaptor protein receptors (SNAP receptors, SNAREs) mediate fusion between vesicles and target membranes and are classed as Q‐ or R‐SNAREs based on their sequence. We analysed gain‐ and loss‐of‐function mutants, dominant‐negative transgenics and localisation of the Arabidopsis R‐SNARE VAMP714 protein to understand its function.
- We demonstrate that VAMP714 is essential for the insertion of PINs into the plasma membrane, for polar auxin transport, root gravitropism and morphogenesis. *VAMP714* gene expression is upregulated by auxin, and the VAMP714 protein co‐localises with endoplasmic reticulum and Golgi vesicles and with PIN proteins at the plasma membrane.
- It is proposed that VAMP714 mediates the delivery of PIN‐carrying vesicles to the plasma membrane, and that this forms part of a positive regulatory loop in which auxin activates a VAMP714‐dependent PIN/auxin transport system to control development.

## Introduction

The polarity of eukaryotic cells is associated with diverse aspects of cell differentiation and development, and one feature of this is the polar distribution of membrane proteins, to promote directional signalling or transport of molecules or ions. In plants, local biosynthesis and the regulated polar transport of auxin contribute to the generation of auxin gradients within tissues, necessary for spatially regulated gene expression and development (Reinhardt *et al*., 2000; Petrášek & Friml, 2009; Vanneste & Friml, 2009). Members of the PIN‐FORMED (PIN) family of auxin efflux carriers accumulate in the plasma membrane on specific sides of the cell and determine the direction of auxin flow through tissues (Wiśniewska *et al*., 2006; Vieten *et al*., 2007).

Rapid changes in cell polarity involve clathrin‐mediated endocytosis of PINs, dependent on both ARF‐GEF (guanine‐nucleotide exchange factors for ADP‐ribosylation factor GTPases)‐ and Rab5 GTPase‐dependent recycling (Steinmann *et al*., 1999; Geldner *et al*., 2001; Kleine‐Vehn *et al*., 2008; Kitakura *et al*., 2011). Auxin itself inhibits this recycling, resulting in an accumulation of PIN proteins at the plasma membrane, so promoting its own efflux (Paciorek *et al*., 2005). While the endocytic model accounts for the dynamic mobilisation of PINs to different surfaces of the cell, it does not explain mechanistically how PIN proteins are delivered to the plasma membrane following their translation in the endoplasmic reticulum (ER).

Eukaryotes have evolved *N*‐ethylmaleimide‐sensitive factor adaptor protein receptors (SNAP receptors, SNAREs) as mediators of fusion between vesicular and target membranes. SNAREs can be grouped as Q‐ and R‐SNAREs based on the occurrence of either a conserved glutamine or arginine residue in the centre of the SNARE domain (Fasshauer *et al*., 1998). In Arabidopsis, Vesicle‐Associated Membrane Protein7 (VAMP7)‐like R‐SNAREs fall into two gene families; four VAMP71 group proteins are involved in endosomal trafficking (Uemura *et al*., 2004; Hong, 2005) and eight VAMP72 group proteins are involved in secretion (Sanderfoot, 2007; Zhang *et al*., 2015). VAMPs have roles in abiotic stress tolerance (VAMP711, VAMP712; Leshem *et al*., 2010; Xue *et al*., 2018), in gravitropic responses (Yano *et al*., 2003), in cell plate formation (VAMP721, VAMP722; Zhang *et al*., 2011; Karnik *et al*., 2013; EI‐Kasmi *et al*., 2013; Yun *et al*., 2013; Zhang *et al*., 2017; Uemura *et al*., 2019), in cytokinesis (Collins *et al*., 2003; Karnik *et al*., 2013), in defence responses (Kwon *et al*., 2008; Zhang *et al*., 2011, 2017), and in the transport of phytohormones (Dacks & Doolittle, 2002; Enami *et al*., 2009).

We identified a gain‐of‐function mutant of *VAMP714* following an activation tagging screen in Arabidopsis (Casson & Lindsey, 2006). VAMP714 is structurally related to VAMPs 711, 712 and 713, and previous data indicated that, while GFP fusions with VAMP711, 712 and 713 localise to the vacuole in Arabidopsis suspension culture protoplasts, GFP‐VAMP714 co‐localises with the Golgi marker VENUS‐SYP31 (Uemura *et al*., 2004), but its function is unknown. In this paper, we describe a combination of genetics, transgenics and cell biological approaches to investigate the function of VAMP714.

## Materials and Methods

### Plant materials

Wild‐type *Arabidopsis thaliana* plants (ecotype Col‐0) and activation tagging populations (Casson & Lindsey, 2006) and growth conditions (Casson *et al*., 2009) have been described previously. We identified two loss‐of‐function mutants of *VAMP714* from the SALK and GABI‐Kat collections of T‐DNA insertion mutants (SALK_005917 and GABI_844B05; www.signal.salk.edu), obtained from the Nottingham Arabidopsis Stock Centre (Nottingham University, Sutton Bonington, UK). RT‐PCR analysis showed that neither mutant expresses the *VAMP714* gene to detectable levels (Supporting Information Fig. S1a). PCR was used to identify homozygous insertion mutants among the GABI_844B05 F1 plants, using oligonucleotide primers to amplify the *VAMP714* gene from wild‐type but not from insertion lines: 5′‐CTGTTGTAGCGAGAGGTACCG‐3′ and 5′‐ AAGCATGTCAACAAGACCCTG‐3′. To confirm T‐DNA insertion sites, a *VAMP714* primer (5′‐AAGCATGTCAACAAGACCCTG‐3′) and a T‐DNA left border primer (5′‐ATATTGACCATCATACTCATTGC‐3′) were used to amplify the T‐DNA flanking sequence.

Genetic crosses between Arabidopsis plants were made under a Zeiss STEMI SV8 dissecting stereomicroscope (Carl Zeiss Ltd., Welwyn Garden City, Herts, UK) as described (Souter *et al*., 2002). Arabidopsis seeds transgenic for the marker QC25 and *DR5::GUS* were kindly provided by Professor Ben Scheres (Wageningen University).

For hormone/inhibitor treatments of seedlings grown *in vitro*, *proVAMP714*::*GUS* seedlings were germinated aseptically on growth medium and at 7 d post germination (dpg) were transferred to growth medium containing auxin (indole‐3‐acetic acid, IAA) and, for comparison, cytokinin (benzylaminopurine, BAP), the ethylene precursor ACC or the polar auxin transport inhibitor 2,3,5‐triiodobenzoic acid (TIBA) for a further 5 d before analysis. For drug treatments, 5‐d‐old seedlings were incubated in half‐strength Murashige and Skoog (½MS) liquid medium supplemented with 50 µM brefeldin A (50 mM stock in DMSO; Sigma‐Aldrich), and 20 µM latrunculin B (20 mM stock in DMSO; Sigma‐Aldrich). DMSO in the same final concentration (0.1%) was added to the negative controls. Each treatment for confocal imaging was repeated at least three times with similar results.

### Gravitropism assays

Mutant and wild‐type seedlings were grown on standard agar plates for 4 d and turned to a 90° angle to measure the angle of bending towards gravity. The angle towards the gravity was measured after 8, 12 and 24 h. The curvature of 20 seedlings for each genotype was determined.

### Gene constructions, plant transformation and transient gene expression

To create dominant‐negative mutant proteins, we expressed a nonfunctional fragment of the VAMP714 protein expected to bind to the Qa, Qb and Qc complex of SNARE and inhibit the binding of the native protein (Tyrrell *et al*., 2007). For constructing the dominant‐negative gene construct, the Longin and SNARE domains of the *VAMP714* gene sequence were amplified without the transmembrane domain, using the oligonucleotide primers 5′‐GGGGACAAGTTTGTACAAAAAAGCAGGCTTCGTTGTAGCGAGAGGTACCGTG‐3′, and 5′‐GGGGACCACTTTGTACAAGAAAGCTGGGTCCTATTAGCATTTTTCATCCAAAG‐3′. The amplified sequence was cloned directly into the pCR^®^2.1‐TOPO vector (Invitrogen, Paisley, UK) and then as an *Eco*RV fragment into the pDNOR207 Gateway entry vector and then pMDC43 destination vector (Invitrogen, Paisley, UK), under the transcriptional control of the CaMV35S gene promoter. qRT‐PCR showed that the relative abundance of the truncated transcript of *VAMP714* was higher in two independent dominant‐negative transgenic lines than is the native transcript in Col‐0 wild‐type plants (Fig. S1e). T4 transgenics were produced by selfing, and at least 10 independent lines were analysed phenotypically.

To amplify the *VAMP714* promoter, the following oligonucleotide primer pairs were used: 5′‐GTCGAGCAGAGATCCTAGTTAGTGAGTCC‐3′ and 5′‐GTCGAGGTGATTCGATGACAGAGAGTGGAG‐3′; the promoter PCR product was cloned into pCR^®^2.1‐TOPO and then as a *Sal*1 fragment into promoterless GUS reporter binary vector pΔGUSCIRCE for *proVAMP714::GUS*.

For the *pro35S::VAMP714:GFP* fusion protein and the *pro35S::VAMP714* constructs, the coding region was amplified using primers 5′‐TTAATTAACGCGATTGTCTATGCTGTTGTAGCG‐3′ and 5′‐CAGATTTTAAGATCTGCATGATGG‐3′, and the product was cloned into the pBIN‐GFP vector (Dr David Dixon, Durham University). For *proVAMP714::VAMP714:CFP* and *proVAMP714::VAMP714:mCherry*, a *c*. 3.5 kb genomic fragment, comprising *c*. 2 kb promoter and 1.5 kb coding sequence of the *VAMP714* gene, was amplified using primers 5′‐GGGGACAAGTTTGTACAAAAAAGCAGGCTCAGAGATCCTAGTTAGTGAGTCC‐3′ and 5′‐GGGGACCACTTTGTACAAGAAAGCTGGGTCAGATCTGCATGATGGTAAAGTG‐3′. The PCR product was cloned into pCR^®^2.1‐TOPO vector and then as an *Eco*RV fragment into the pDNOR207 Gateway entry vector and then pGHGWC destination vector. All constructs were validated by sequencing. Expression of the *pro35S::VAMP714* in VAMPOx transgenic plants was confirmed in independent lines by qRT‐PCR (Fig. S1e).

Transgene plasmids were introduced into *Agrobacterium tumefaciens* C58C3 by triparental mating, and plant transformation was performed by the floral dip method (Clough & Bent, 1998). Transformed plants were selected using standard growth medium supplemented with kanamycin (50 µg ml^−1^ for *proVAMP714::GUS*), Basta (15 µg ml^−1^ for *pro35S::VAMP714:GFP*) or hygromycin (50 µg ml^−1^ for *proVAMP714::VAMP714:CFP* and *proVAMP714::VAMP714:mCherry*).

Transient expression of *pro35S*::*VAMP714:GFP* and *ST–RFP* constructs was carried out in onion epidermal peels following microprojectile bombardment using the Helios Gene Gun system (Bio‐Rad Laboratories, Hemel Hempstead, UK), or in leaves of *Nicotiana benthamiana*. Plates containing bombarded onion sections were covered with aluminium foil and incubated at 22°C overnight, after which the inner layer of the onion tissue was peeled off carefully and mounted on a glass slide with drop of water, covered with a coverslip and viewed under a Leica SP5 Laser Scanning Microscope (Leica Instruments, Heidelberg, Germany). Transient expression and protein co‐localisation in *N. benthamiana* was carried out following injection of *Agrobacterium tumefaciens* containing *pro35S*::*VAMP714:mCherry* and *pro35S::PIN1:GFP* as described previously (Sparkes *et al*., 2006).

### Gene expression analysis

Localisation of GUS enzyme activity in transgenic plants containing the *proVAMP714::GUS* reporter gene was performed as described (Short *et al*., 2018). Stained samples were fixed in Karnovsky’s fixative (4% paraformaldehyde and 4% (v/v) glutaraldehyde in 0.1 M phosphate buffer), dehydrated in an ethanol series and embedded in LR White resin (Historesin™ Embedding Kit, Leica Instruments, Heidelberg, Germany) before sectioning, as described (Topping *et al*., 1997).

For transcript analysis, RNA was extracted from seedlings 7 dpg using the RNeasy Plant RNA Extraction kit (Qiagen Ltd, Surrey, UK). RT‐PCR was performed using the OneStep RT‐PCR kit (Qiagen) as per the manufacturer’s instructions. Oligonucleotide primer pairs used for amplification of *VAMP714* were: 5′‐GTCGAGCAGAGATCCTAGTTAGTGAGTCC‐3′ and 5′‐GTCGAGGTGATTCGATGACAGAGAGTGGAG‐3′ primers. The *ACTIN2* gene was used as a positive control, using primers 5′‐GGATCGGTGGTTCCATTCTTGC‐3′ and 5′‐AGAGTTTGTCACACACAAGTGCA‐3′.

For quantitative RT‐PCR, the following primers were used: for *VAMP714*, 5′‐GAGATTCGATCGGTCATGGT‐3′ and 5′‐GGTAAAGTGATTCCTCCG‐3′; for *VAMP713*, 5′‐TTGTGAAAACATATGGCCGA‐3′ and 5′‐CTAGCAACTCCAAACGCTCC‐3′; for *VAMP712*, 5′‐AACGTACTGATGGCCTCACC‐3′ and 5′‐ATGTTCGCGGTTTTATCGAC‐3′; for *VAMP711*, 5′‐GGTGGAGAAACTGCAAGCTC‐3′ and 5′‐ACACACTTCGCAAAGCAATG‐3′; for *IAA1*, 5′‐GGAAGTCACCAATGGGCTTA‐3′ and 5′‐GAGATATGGAGCTCCGTCCA‐3′; and for *IAA2*, 5′‐CACCAGTGAGATCTTCCCGT‐3′ and 5′‐AGTCTAGAGCAGGAGCGTCG‐3′.

### Auxin transport assays

Basipetal shoot auxin transport assays were carried out as described (Chilley *et al*., 2006). 2.5 cm of inflorescence stem segments lacking branches were excised and the apical (upper) end placed in 20 µl MS salts medium in Eppendorf tubes. This pretreatment prevents air bubbles entering the auxin transport system. Stem segments were then transferred to fresh tubes containing medium supplemented with 0.08 µCi ml^−1^ ^3^H‐IAA (c. 3.5 µM IAA), again with the apical ends in the liquid medium. Samples were incubated for 18 h before the basal 5 mm of the sample was removed and placed in 4 ml of scintillation fluid, and incubated for 48 h before scintillation. Noninverted samples (in which the basal ends were placed in the medium) were included to control for nonspecific transport. Samples incubated in nonradioactive medium were used to detect baseline activity or radioactive contamination.

Acropetal root auxin transport assays were carried out on 2 dpg Arabidopsis seedlings. Agar blocks (1% w/v, 2–3 mm wide) were prepared containing 500 nM ^3^H‐IAA (specific activity is 5.75 µCi ml^−1^; GE Amersham, UK) plus 10 µM IAA in 1% v/v DMSO. The ^3^H‐IAA blocks were placed onto the top of roots just below the root–shoot junction. For each root analysed, the distance between the application site and the root tip was constant; the plants were inverted and left for 1 h per cm. The distal 5 mm at the root tip was removed and the sample transferred to 4 ml scintillation fluid (EcoScint A, National Diagnostics) and incubated for 48 h before measuring in the scintillation counter.

All data were expressed as disintegrations per minute (DPM). Results represent the means of five independent assays ± SD.

### Protein localisation and confocal microscopy

PIN protein immunolocalisation was carried out as described (Short *et al*., 2018). Fluorescence levels were quantified using Imagej software (National Institutes of Health, http://rsb.info.nih.gov/ij). At least three independent analyses were carried out, and for each, six random samples for each of 10 roots were measured, using identical confocal settings for each analysis. Results are presented as means ± standard deviation. We thank Professor Klaus Palme (University of Freiburg) for kindly donating PIN antibodies. Confocal imaging used a Leica SP5 Laser Scanning Microscope (Leica, Heidelberg, Germany). Light microscopy was performed on a Zeiss Axioscop microscope (Carl Zeiss Ltd, Welwyn Garden City, UK) with the DIC/Nomarski optics or an Olympus SZH10 microscope system (Olympus Microscopes, Southend‐on‐Sea, UK) and *c*. 15 seedlings for each genotype were analysed by QC25‐GUS expression (Sabatini *et al*., 2003) and by lugol staining and cleared and mounted in glycerol (Souter *et al*., 2002).

## Results

**Fig. 1:**
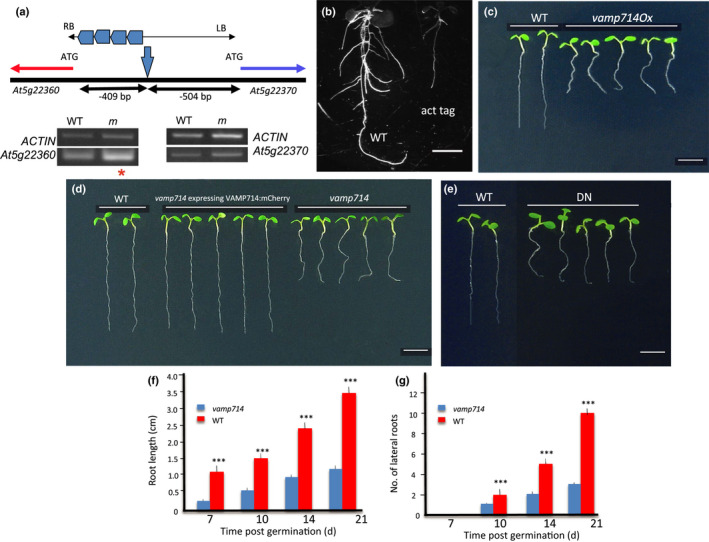
The Arabidopsis *VAMP714* gene is required for correct seedling development. (a) Diagrammatic representation of the activation tag locus, showing the position of the activation tag T‐DNA and the expression analysis of the *At5g22360* and *At5g22370* genes relative to the *ACTIN2* and Col‐0 wild‐type (WT). RB and LB indicate the borders of the T‐DNA insertion element, encompassing enhancer regions (the four pentagons) and showing the site of insertion. The distances from the T‐DNA insertion site to the transcriptional start codon of each of the adjacent genes are indicated. Red asterisk indicates enhanced expression of *At5g22360* (*VAMP714*) in the mutant (m), compared with in the wild‐type (WT). (b) Wild‐type and activation‐tagged *VAMP714* overexpressing (act tag) seedlings at 14 d post germination (dpg). Bar, 5 mm. (c) Seedlings (7 dpg) of wild‐type, *vamp714* mutant and *vamp714* mutant transformed with a *proVAM714::VAMP714:mCherry* gene fusion, showing functional complementation of the mutant by the fusion gene. Bar, 5 mm. (d) Seedlings (7 dpg) of wild‐type and *pro35S::VAMP714:GFP* transgenic overexpressers. Bar, 5 mm. (e) Seedlings (7 dpg) of wild‐type and VAMP714 dominant‐negative mutant transgenics. Bar, 5 mm. (f) Primary root length of wild‐type and *vamp714* loss‐of‐function mutants grown on vertical agar plates over 21 dpg. Mean of 20 replicates ± standard error of the mean. ***, *P* < 0.001, Student's *t‐*test. (g) Lateral root number of wild‐type and *vamp714* loss‐of‐function mutants grown on vertical agar plates over 21 dpg. Mean of 20 replicates ± standard error of the mean SE. ***, *P* < 0.001, Student's *t‐*test.

We used an activation tagging screen (Casson & Lindsey, 2006) to identify Arabidopsis mutants defective in root development, and one was associated with the upregulation of gene At5g22360, encoding the 221 amino acids VAMP714 protein, this gene was then the focus of further studies (Fig. 1a). To confirm a potential role of the *VAMP714* gene in root development, two independent loss‐of‐function insertional mutants were identified from the SALK and GABI‐Kat collections of T‐DNA insertion mutants (SALK_005917 and GABI_844B05; www.signal.salk.edu), and a dominant‐negative mutant was constructed (Fig. S1). PCR‐based genotyping was used to confirm the sites of T‐DNA insertion in the SALK and GABI‐Kat lines, and to confirm loss of *VAMP714* expression (Fig. S1a). In the SALK mutant the T‐DNA was located in the first intron, and in the GABI‐Kat mutant the T‐DNA was located in the third exon. The dominant‐negative gene construct was designed to comprise the Longin and SNARE domains but lacking the transmembrane domain, so that it would bind to the Qa, Qb and Qc complex of SNARE but inhibit the binding of the native protein (Tyrrell *et al*., 2007; Fig. S1b).

**Fig. 2:**
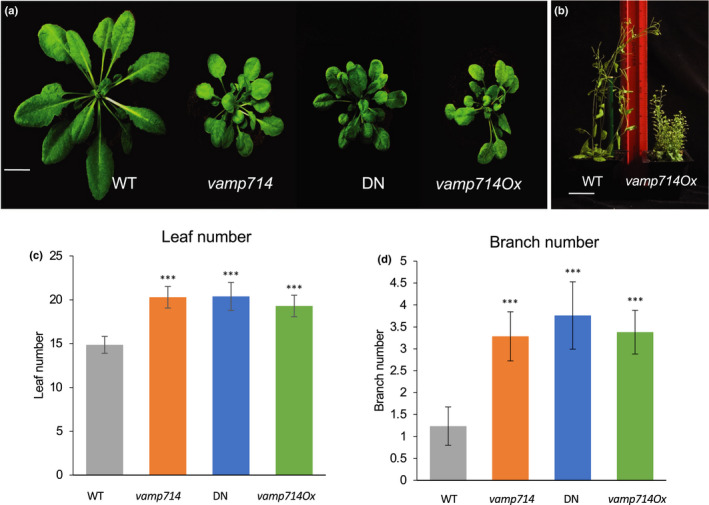
The Arabidopsis *VAMP714* gene is required for correct shoot architecture. (a) Shoot phenotypes of wild‐type (WT), *vamp714* mutant, VAMP714 dominant‐negative mutant (DN) and transgenic *VAMP714* overexpressers (VAMPOx) seedlings at 4 wk post germination. Bar, 1 cm. (b) Wild‐type (WT, L) and transgenic *pro35S:VAMP714* plants (*vamp714Ox*, R) plants at 8 wk post germination. Bar, 3 cm. (c) Rosette leaf number of wild‐type, *vamp714* mutant, VAMP714 DN and transgenic *VAMP714* overexpressers (VAMPOx) seedlings at 4 wk post germination. Error bars represent the SD of the mean of three biological replicates. ***, significant difference at *P* < 0.005, Student's *t*‐test. (d) Shoot branch number of wild‐type, *vamp714* mutant, VAMP714 DN and transgenic *VAMP714* overexpressers (VAMPOx) seedlings at 8 wk post germination. Error bars represent SD of the mean of three biological replicates. ***, *P* < 0.005, Student's *t*‐test.

Seedlings of both *vamp714* mutants each showed very similar phenotypes, being smaller than wild‐type with reduced root systems (Fig. 1b–g). The mutant phenotype was functionally complemented by a *proVAMP714::VAMP714:mCherry* transgene (Fig. 1c), showing that the correct gene had been identified, corresponding to the phenotype. Expression of the transgene was demonstrated by confocal microscopy (Figs 4h,8a, see later), confirming transformation. By 21 dpg, *vamp714* insertional mutants grown in soil developed a shorter primary root than wild‐type (1.2 ± 0.2 cm vs 3.5 ± 0.5 cm, *n* = 20; Fig. 1f), with fewer lateral roots (3.1 ± 1.0 vs 10.0 ± 2.0, *n* = 20; Fig. 1g) although this represents only a slightly reduced lateral root density (a mean of 2.6 lateral roots per cm at 21 dpg for *vamp714* compared with 2.8 for wild‐type). Both transgenic *VAMP714* overexpressers (Fig. 1d) and dominant‐negative mutants also showed a reduced seedling root system (Fig. 1e), with a mean primary root length of 1.8 ± 0.2 cm (*n* = 20) at 21 dpg, and mean lateral root numbers of 3.2 ± 0.9, *n* = 20. The mutant and overexpressing seedlings also showed a reduced hypocotyl length compared with wild‐type (Fig. S2a–f). Compared with wild‐type, the *vamp714* loss‐of‐function and dominant‐negative mutants, and transgenic overexpressors, each showed a dwarfed and excessive leaf and shoot branching phenotype (Fig. 2a–d). The common phenotypes between diverse mutant lines, and the genetic complementation studies, show that the observed developmental abnormalities were due to the disruption of expression of the *VAMP714* gene rather than to other mutations.

**Fig. 3:**
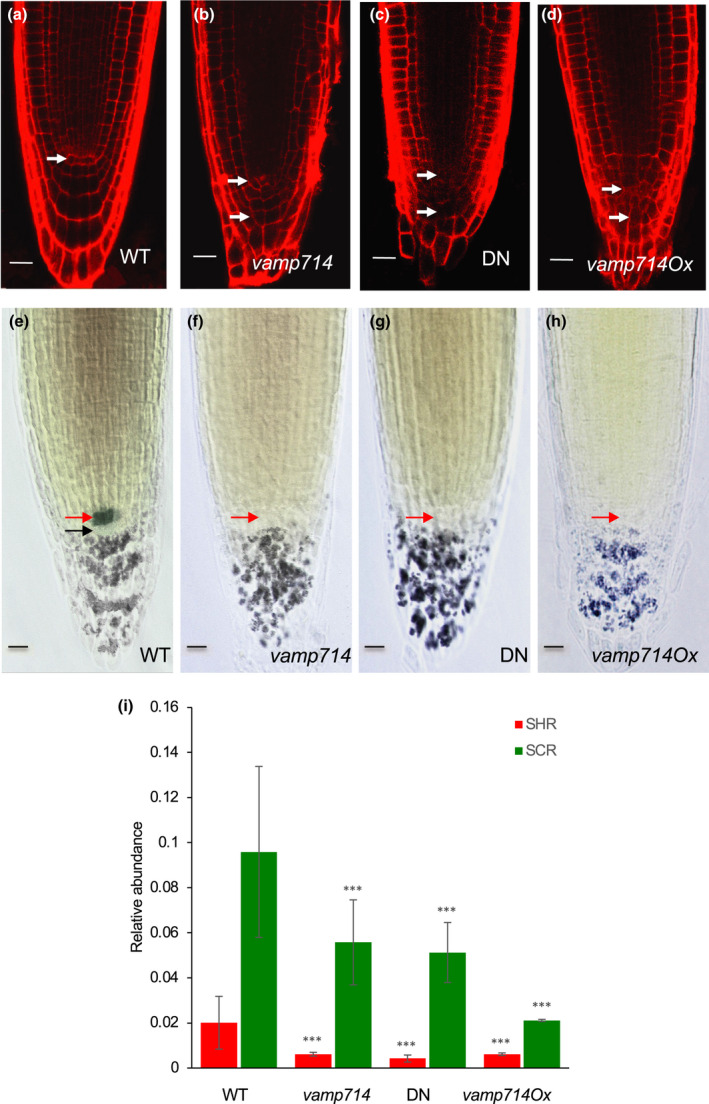
Functional VAMP714 is required for correct root cell patterning, QC maintenance and meristem gene expression in Arabidopsis. (a–d) Confocal imaging of root tips of (a) wild‐type (WT), (b) *vamp714* mutant, (c) VAMP714 dominant‐negative mutant (DN) and (d) transgenic VAMP714 overexpressing (vamp714Ox) seedlings (7 d post germination, dpg) stained with propidium iodide. (a) White arrow indicates position of the QC cells. (b–d) White arrows highlight examples of abnormal cell shapes. Representative images from *c*. 15 seedlings for each genotype. Bars, 10 µM. (e–h) Double labelling of QC and differentiated columella cells, visualised by QC25 GUS expression (showing the QC) and amyloplast (lugol) staining in the columella cells of wild‐type (a) and mutant or overexpressing seedlings (f–h) at 7 dpg. The mutant and overexpressing lines show no QC25 expression (approximate expected position indicated by red arrows). Black arrow in (e) indicates the starch‐free columella stems cells below the QC. Representative images from *c*. 15 seedlings for each genotype. Bars, 10 µM. (i) qRT‐PCR analysis of mRNA abundance of the QC identity genes *SHR* and *SCR* in wild‐type, *vamp714* mutants, DN and transgenic *VAMP714* overexpressers (*vamp714Ox*), compared with *ACTIN2* expression at 7 dpg. Data represent the means of three biological replicates ± SD. Significant differences between wild‐type, dominant‐negative, loss‐of‐function mutant and overexpressers were found for each gene: expression at *P* < 0.001 (***) are indicated, ANOVA, with significance at *P* = 0.0008 (SHR, *vamp714*); *P* = 0.0003 (SHR, DN); *P* = 0.0008 (SHR, *vamp714Ox*); *P* = 0.0009 (*vamp714*, SCR); *P* = 0.0002 (DN, SCR); *P* = 1.01E‐07 (SCR, *vamp714Ox*).

To investigate the organisation of the root tip, which is the source of stem cells for cell division in the meristem and ultimately root growth, we used a combination of imaging and gene expression analysis. Propidium iodide (PI) is a fluorescent dye that is excluded by intact cells, and can be used to visualise cell patterning using confocal microscopy (e.g. Rowe et. al., 2016). PI staining at 7 dpg reveals a more disorganised tissue patterning in *vamp714* mutant roots compared with Col‐0 (Fig. 3a–d). Wild‐type columella cells are relatively elongated in the apical–basal axis, giving rise (in Col‐0) to normally four tiers (Fig. 3a), while the mutants have similar numbers but less distinct tiers, with less regular columella cell shapes (Fig. 3b–d). Lugol, which stains starch, similarly showed an abnormal patterning of amyloplast‐containing columella cells in *vamp714* mutant roots and less discrete columella tier delineation that is seen in the wild‐type at 7 dpg (Fig. 3e–h). The QC25 marker gene is specifically expressed in the quiescent centre (QC) of the root meristem, and the QC regulates the stem cell identity of the surrounding initials, which contribute to columella development (Sabatini *et al*., 2003). The identity of the QC is itself regulated by auxin‐mediated PLT transcription factor expression (Aida *et al*., 2004), and by the expression of the GRAS transcription factors SHORT‐ROOT (Helariutta *et al*., 2000) and SCARECROW (SCR) (Sabatini *et al*., 2003); and control of the organisation of the columella pattern is auxin‐dependent (Sabatini *et al*., 1999). *vamp714* mutants lack an appropriately specified QC as seen by the lack of QC25 expression, and additional layers of (disorganised) starch‐containing columella stem cells suggest a failure of QC and columella stem cell activity (Fig. 3e–h). To further investigate stem cell gene expression in these plants, we measured the transcription of the genes *SHR* and *SCR* at 7 dpg by qRT‐PCR. The transcript levels of both genes were reduced in *vamp714* insertional mutants, dominant‐negative mutants and overexpressers, consistent with the loss of identity of QC cells and possibly of other stem cells in which these genes are expressed (Fig. 3i). These observations are consistent with the observed short‐root phenotype of the *vamp714* mutants.

**Fig. 4:**
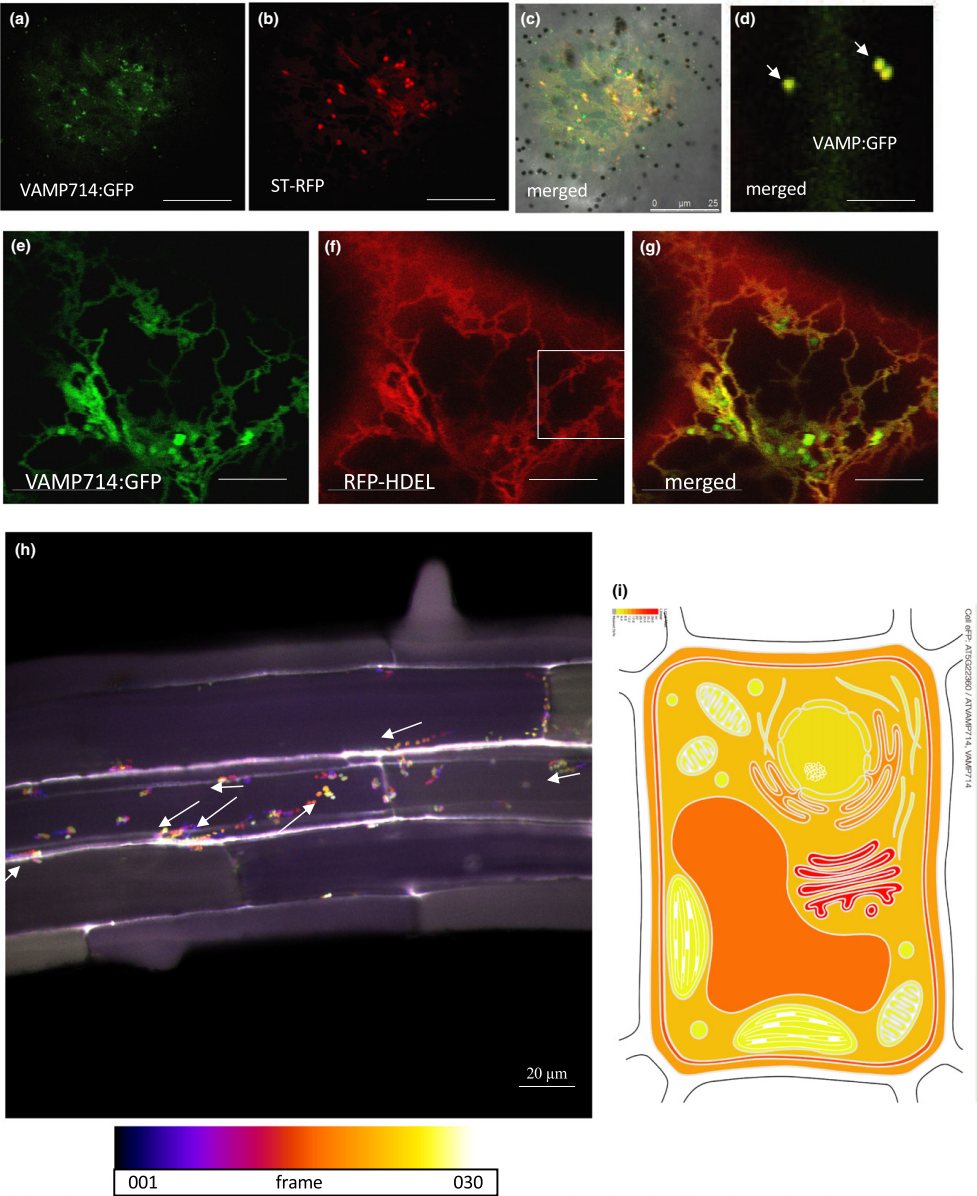
VAMP714 fusion proteins localise to vesicles in Arabidopsis. (a–d) Transient expression and localisation of VAMP714:GFP (a) and the Golgi membrane marker ST–RFP (b), showing co‐localisation in merged images (c, d; arrowheads indicate individual vesicles showing co‐localisation). Bars, 25 µm (a–c), 10 µm (d). (e–g) Transient expression and localisation of VAMP714:GFP (e) and the endoplasmic reticulum (ER) membrane marker RFP‐DEL (f), showing some co‐localisation in merged images (g). Bars, 15 µm. (h) Still image captured from a video (see Supporting Information Video S1 for video sequence) with temporal‐colour code tracking of VAMP714:mCherry‐positive vesicle movement. Lighter colours represent greater movement over time, reflected in the higher frame number of the video. White arrows indicate the direction of vesicle transport to the plasma membrane. Bar, 20 µm. (i) Heatmap of predicted VAMP714 subcellular location, using online tool at http://bar.utoronto.ca/eplant/, showing highest levels (red) at the Golgi, ER and plasma membrane.

Consistent with the predicted role as a vesicle‐associated membrane protein, a VAMP714:GFP fusion (under the transcriptional control of the *CaMV35S* gene promoter) was constructed for testing *in vivo*, and found to locate to vesicles. Given that a VAMP714:mCherry fusion protein is functional, as demonstrated by genetic complementation (Fig. 1c), we expect a VAMP714:GFP fusion to similarly be functional. Stably transformed Arabidopsis plants were screened to identify lines that express relatively low levels of *pro35S::VAMP714:GFP* to avoid GFP protein aggregation, and both stable transformants and transiently transformed onion epidermal peels show GFP signal in discrete vesicles, with additional plasma membrane localisation seen in the stable transformants (Fig. 4). The vesicles were identified in onion epidermal cells as Golgi by co‐labelling with the Golgi membrane marker ST–RFP (sialyltransferase–red fluorescent protein, Runions *et al*., 2006; Fig. 4a–d). The majority of ST‐RFP‐positive vesicles co‐localised with VAMP714:GFP‐positive vesicles (98.3%, *n* = 45 cells), but a fewer proportion of VAMP714:GFP‐positive vesicles co‐localised with ST‐RFP‐positive vesicles (52.1%, *n* = 45 cells), indicative of VAMP714 being found in other subcellular compartments. Transient expression studies also revealed co‐localisation with the endoplasmic reticulum‐targeted red fluorescent protein RFP‐HDEL (Lee *et al*., 2013; Fig. 4e–g) and at the plasma membrane (Fig. 4e–g). We showed by video confocal microscopy in stable Arabidopsis plants that the VAMP714:GFP‐positive vesicles are mobile (Fig. 4h; Video S1). These data are consistent with computational and previous experimental predictions of subcellular location in Arabidopsis (Uemura *et al*., 2004; Fig. 4i).

The spatial expression pattern of the *VAMP714* gene was examined in seedlings expressing a promoter reporter fusion (*proVAMP714*::*GUS)* using histochemical localisation of GUS activity (nine independent transgenic lines showed similar patterns of GUS activity; representative images are shown in Fig. S3a–e). GUS activity was detected in roots, and most strongly in vascular tissues of primary and lateral roots, although also at lower levels in the root cortex and in the QC; and at relatively low levels in cotyledon veins, but not in leaf. This expression pattern is consistent with data from the analysis of the transcriptomes of individual root cell types in day 6 seedlings (Birnbaum *et al*., 2003; Nawy *et al*., 2005; and visualised at www.bar.utoronto.ca; Fig. S3f).

As primary and lateral root growth, correct columella patterning, and shoot branching control are dependent on correct auxin transport and/or signal transduction, and *VAMP714* is expressed in roots and vascular tissues that have relatively high auxin responses (Sabatini *et al*., 1999; Peret *et al*., 2009), these observations led us to hypothesise a role for *VAMP714* in mediating auxin signalling.

**Fig. 5:**
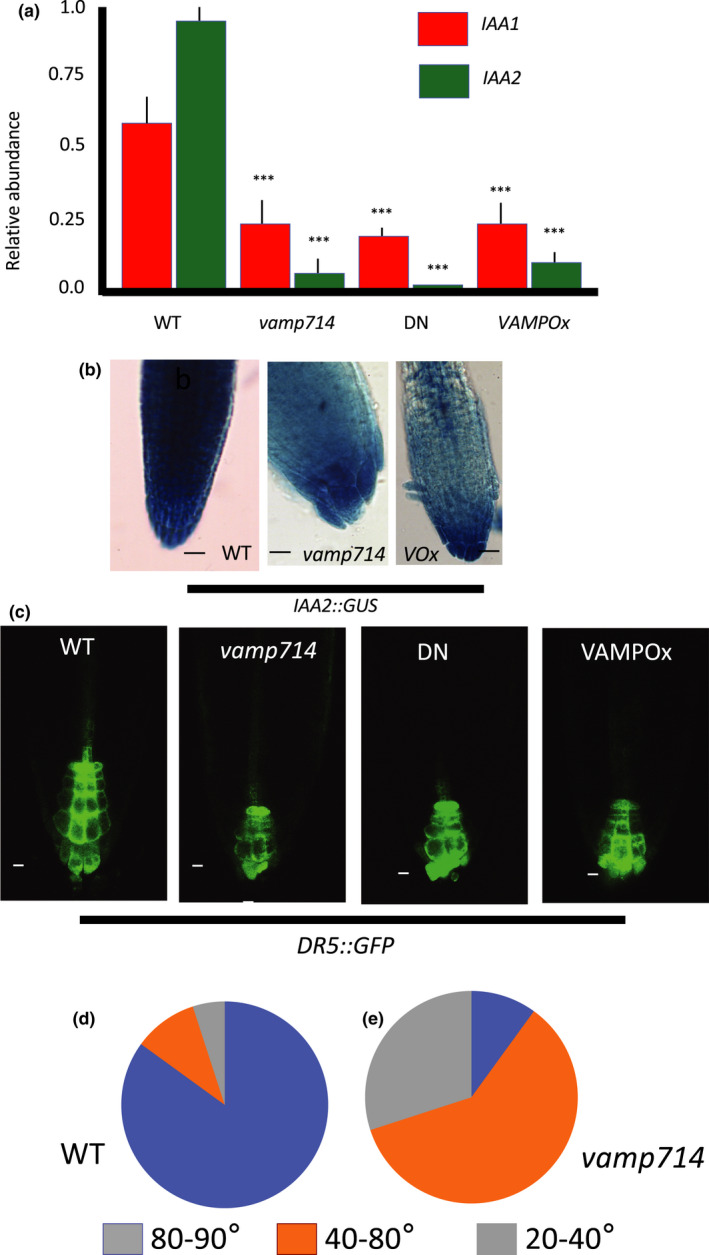
Arabidopsis VAMP714 is required for correct auxin‐mediated gene expression and root gravitropism. (a) qRT‐PCR analysis of mRNA abundance for the auxin‐inducible *IAA1* and *IAA2* genes in wild‐type (WT), *vamp714* mutant, VAMP714 dominant‐negative mutant (DN) and transgenic overexpressing (VAMP Ox) seedlings at 7 d post germination (dpg), compared with *ACTIN2* expression. ***, *P* < 0.005, Student’s *t*‐test, error bars indicate SD of four biological replicates. (b) *proIAA2::GUS* reporter activity in wild‐type (b), *vamp714* mutant (c) and transgenic overexpressing (VOX, d) roots at 7 dpg. Bars, 10 µm. (c) *proDR5::GFP* expression in wild‐type, *vamp714* mutant, transgenic dominant‐negative VAMP714 mutant (DN) and transgenic overexpressing (VAMPOx) roots at 7 dpg. Bars, 10 µm. (d, e) Diagrammatic representation of the gravitropic responses of wild‐type (d) and *vamp714* mutants (e) at 24 h after shifting the vertical axis by 90°. The pie‐charts indicate the proportion of seedlings showing bending responses at between 80° and 90° from horizontal (blue), 40° to 80° from horizontal (orange) and 20° to 40° from horizontal (grey).

To investigate auxin responses in mutant and overexpressing plants, we measured the transcription of the auxin‐regulated genes *IAA1* and *IAA2* (Hagen & Guilfoyle, 2002) at 7 dpg by qRT‐PCR. The transcript levels of both genes were found to be reduced compared with wild‐type in *vamp714* insertional mutants, dominant‐negative mutants and also in *VAMP714* overexpressors (Fig. 5a). Histochemical analysis of the auxin reporter genes *IAA2::GUS* (Swarup *et al*., 2001) and *DR5::GFP* (Sabatini *et al*., 1999) revealed altered expression in the *vamp714* mutants and overexpressor (Fig. 5b,c). Compared with wild‐type, *IAA2::GUS* staining is more diffuse and distally shifted to the disorganised columella of both *vamp714* mutant and overexpressing seedlings (Fig. 5b); while *DR5::GFP*, which is mainly detected in the QC and columella in the wild‐type, exhibits a broadly similar spatial pattern in the roots of the mutants and overexpressers to wild‐type but reveals the disorganised cellular patterning of the mutants and overexpressers and distal shift in expression (Fig. 5c). These data are indicative of incorrect auxin distribution or auxin content in the root tip and demonstrate that wild‐type *VAMP714* expression is required for correct auxin distribution and responses. Gravitropism is an auxin‐mediated response and linked to correct function of starch‐containing columella cells (Wolverton *et al*., 2011), and in gravitropism assays, only 10% of *vamp714* roots showed a true gravitropic response, compared with 85% of wild‐type roots at 24 h (*n* = 20; Fig. 5d,e). This further supports a role for VAMP714 in auxin transport‐mediated processes.

Given that VAMP714 is required for correct auxin patterning and responses, we considered that it might itself be activated in response to auxin, since for example auxin promotes *PIN* gene expression and PIN protein localisation (Paciorek *et al*., 2005; Heisler *et al*., 2005). To investigate this hypothesis, wild‐type seedlings were transferred to medium containing 10 µM IAA and the steady‐state transcript levels of *VAMP714* were measured after 12, 24 and 36 h. The auxin treatment increased relative transcript abundance for the *VAMP714* gene *c*. three‐fold by 24 h after treatment, compared with an *ACTIN2* internal control gene (Fig. S4a). Related genes *VAMP711, VAMP712* and *VAMP713* showed similar patterns of auxin‐inducibility (Fig. S4b–d).

**Fig. 6:**
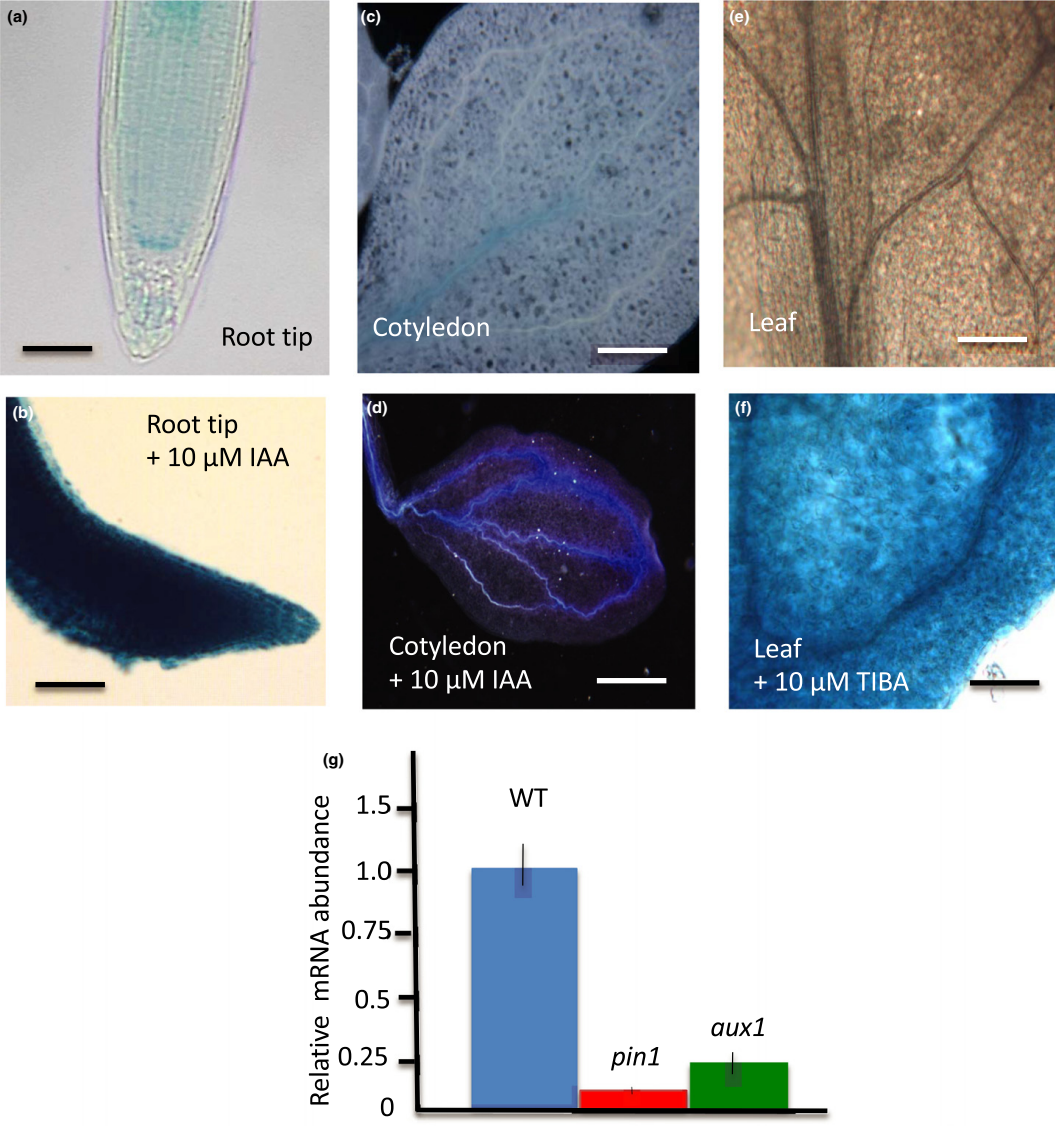
The Arabidopsis *VAMP714* gene is auxin‐regulated. (a, b) Primary root tip of 7 d post germination (dpg) seedling either untreated (a) or treated with 10 µM IAA for 5 d (b). Bars, 25 µm. (c, d) Cotyledon of seedlings either untreated (c) or treated with 10 µM IAA for 5 d (d). Bars, 25 µm (c), 40 µm (d). (e, f) Leaf of seedling either untreated (e) or treated with 10 µM TIBA for 5 d (f). Bars, 25 µm (e), 30 µm (f). (g) qRT‐PCR analysis of mRNA abundance of *VAMP714* in wild‐type, *pin1* and *aux1* mutant seedlings at 7 dpg. Expression levels are relative to *ACTIN2* expression. Data represent means of four biological replicates ± SD.

While exogenous cytokinin and the ethylene precursor 1‐aminocyclopropane‐1‐carboxylic acid (ACC) had no detectable effect on *proVAMP714*::*GUS* expression (data not shown), exogenous auxin (10 µM IAA) induced strong GUS activity in root tips (Fig. 6a,b), and in cotyledon vascular tissues (Fig. 6c,d). Here, 10 µM TIBA treatment, which induces the accumulation of auxin in aerial tissues by inhibition of polar auxin transport, led to an activation of GUS activity in the young leaf (Fig. 6e,f). The observed auxin‐inducibility of expression is consistent with *VAMP714* transcription in vascular and QC cells, which contain relatively high concentrations of auxin (Sabatini *et al*., 1999; Dengler, 2001).

To study the dependence of *VAMP714* expression on correct auxin transport *in planta*, we compared *VAMP714* expression in *pin1* and *aux1* mutants with wild‐type. The *pin1* and *aux1* mutants exhibit reduced polar auxin transport (Okada *et al*., 1991; Bennett *et al*., 1996). In both mutants, the level of *VAMP714* mRNA was significantly reduced compared with wild‐type (Fig. 6g), indicating a requirement for polar auxin transport for the correct expression of *VAMP714*.

**Fig. 7:**
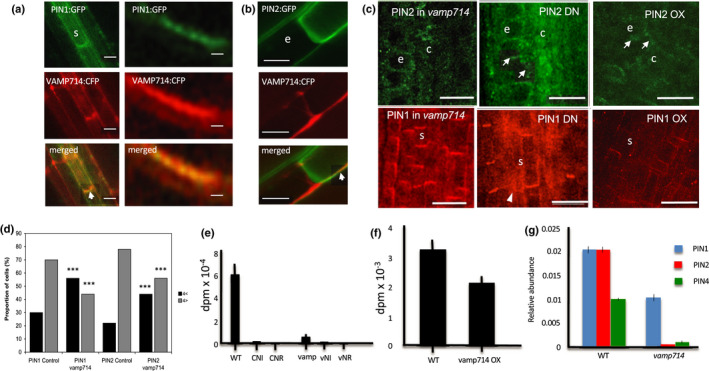
Arabidopsis VAMP714 is required for correct PIN protein localisation and polar auxin transport. (a) proPIN1::PIN1:GFP and (b) proPIN2::PIN2:GFP localisation (upper panels) and co‐localisation with proVAMP714::VAMP714:CFP (lower two panels) at the basal plasma membrane of root cells in transgenic plants. Arrowheads highlight co‐localisation in merged images. ‘s’ indicates stele cell, ‘e’ indicates epidermal cell. (a) Left panels: bars, 10 µm; Bars: (a, left panels), 10 μm; (a, right panels) 1 µm; (b) 10 µm. (c) PIN1 and PIN2 immunolocalisation in seedling roots of *vamp714* mutants (left two panels), dominant‐negative (DN, centre two panels) and *pro35S::VAMP714* overexpressers (OX, right two panels) at 7 d post germination (dpg). Arrowhead exemplifies disrupted PIN localisation. ‘s’ indicates stele cell, ‘e’ indicates epidermal cell, ‘c’ indicates cortex cell. Bars, 20 µm. (d) Quantification of PIN1 and PIN2 distribution in wild‐type and *vamp714* mutant cells, showing the proportion of cells with relatively strong fluorescence signal at the plasma membrane (four‐fold above the cytoplasmic signal and above; grey bars) vs relatively weak signal at the plasma membrane (less then four‐fold above the cytoplasm level; black bars) for wild‐type (control) and mutant (*vamp714*). The mutant exhibits a lower percentage of cells showing the fluorescence signal for both PIN1 and PIN2 at the plasma membrane. ***, Significant difference between wild‐type and mutant at *P* < 0.005, Student's *t*‐test. (e, f) Polar auxin transport measurements in (e) inflorescence stems of *vamp714* mutants and (f) roots of *pro35S::VAMP714* misexpressors. (e) Col‐0 indicates auxin transport in the wild‐type control; CNI is the noninverted wild‐type control (the stem was not inverted, so that the basal region was exposed to the ^3^H‐IAA); CNR is the wild‐type control in nonradiactive medium; vamp indicates auxin transport in the *vamp714* mutant; vNI is the noninverted *vamp714* mutant; vNR is *vamp714* mutant incubated in nonradioactive medium. Data represent the means of five independent assays ± SD. (f) Auxin transport assays in wild‐type (wt) and transgenic *pro35S::VAMP714*‐overexpressing (VAMP Ox) roots. Data represent the means of five independent assays ± SD. (g) qRT‐PCR analysis of mRNA abundance of *PIN1, PIN2* and *PIN4* genes in wild‐type and *vamp714* mutant seedlings, relative to *ACTIN2* expression. Error bars represent means ± SD of three biological replicates.

In view of the diverse auxin signalling‐related defects in *vamp714* mutants and overexpressers, and the prospective role for VAMP714 in membrane vesicles, we investigated a possible role for VAMP714 in PIN localisation and polar auxin transport. In wild‐type cells, PIN1:GFP was localised as expected to the basal membrane of the cells in the central cylinder (Fig. 7a), and PIN2:GFP was localised to the apical membrane of the cells in the root cortex and epidermis (Fig. 7b), as expected. In transgenic plants expressing *proVAMP714::VAMP714:CFP*, both PIN1:GFP and VAMP714:CFP, and PIN2:GFP and VAMP714:CFP, co‐localise at the plasma membrane, though less clearly for PIN2 than for PIN1 (Fig. 7a,b). Co‐localisation between VAMP714:mCherry and PIN1:GFP was confirmed by transient expression and confocal imaging in *Nicotiana benthamiana* leaves (Fig. S5). Immunolocalisation showed that both PIN1 and PIN2 were less concentrated at the plasma membrane in mutant, dominant‐negative and overexpressing plants (Figs 7c,S6). Immunolocalisation of PIN3 and PIN4 similarly show a lack of correct localisation and immunosignal in the region of the columella (PIN3; Friml *et al*., 2002b) and the root stem cell niche (PIN4; Friml *et al*., 2002a), but appeared to be normally localised in the more proximal region of the root meristem, above the QC (Fig. S7). For PIN1 and PIN2, the reduction of localisation at the plasma membrane was accompanied by higher protein levels in the cytoplasm, resulting in lower values of membrane : cytoplasm ratios in the null mutant (Fig. 7d).

To examine the effect of aberrant PIN localisation on auxin transport, we used a [^3^H]‐IAA transport assay. The rate of auxin transport was significantly reduced in both hypocotyl (Fig. 7e) and root (Fig. 7f) of the PIN localisation‐defective *VAMP714* misexpressors, compared with wild‐type controls. Given the proposed regulatory loop in which auxin promotes *PIN* gene expression which then regulates directional auxin transport (Grieneisen *et al*., 2007), we hypothesised that the levels of *PIN* gene expression in the loss‐of‐function *vamp714* might also be reduced. Consistent with this hypothesis, the transcription of *PIN1*, *PIN2* and *PIN4* genes was reduced in the *vamp714* loss‐of‐function mutant (Fig. 7g).

**Fig. 8:**
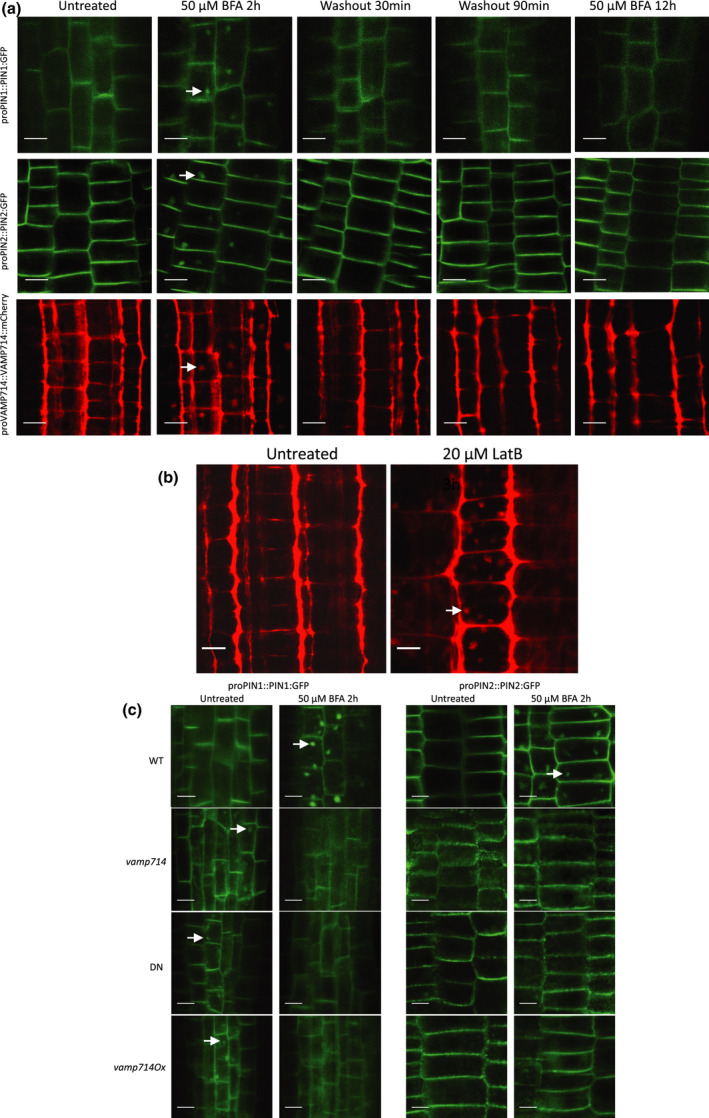
VAMP714, PIN1 and PIN2 exhibit same endosome recycling from BFA compartments to plasma membrane and actin requirements for polar VAMP714 targeting in Arabidopsis. (a) Seedling roots expressing proPIN1::PIN1:GFP, proPIN2::PIN2:GFP and proVAMP714::VAMP714:mCherry were imaged before and after 2 h of treatment with 50 μM brefeldin A (BFA), 30 and 90 min after BFA washout, and after a prolonged 12 h treatment with BFA. The localisation of PIN:GFP proteins and VAMP714:mCherry proteins in the plasma membrane was re‐established by 30 min after washout in the wild‐type. Arrows indicate BFA bodies. Bars, 10 μm. (b) Seedlings expressing proVAMP714::VAMP714:mCherry were imaged before and after 3 h of treatment with 20 μM latrunculin B, revealing sensitivity to actin depolymerisation. Arrow indicates intracellular vesicle accumulation. Bars, 10 μm. (c) Wild‐type (WT), *vamp714* mutant, and VAMP714 dominant‐negative mutant (DN) seedlings expressing either proPIN1::PIN1:GFP or proPIN2::PIN2:GFP imaged before treatment with 50 μM BFA (untreated, left panels) and after 2 h of BFA treatment (right panels). WT seedlings exhibited PIN:GFP internalisation in BFA compartments, whereas *vamp714* mutant and dominant‐negative mutant seedlings showed no PIN accumulation in BFA bodies. Arrows indicate intracellular accumulation of PIN. Bars, 10 μm.

The PIN proteins are dynamically regulated in their subcellular localisation via the endosome recycling pathway (Geldner *et al*., 2001), and we hypothesised that VAMP714‐associated vesicles may also be subject to endosome recycling. This recycling is inhibited by both the vesicle‐trafficking inhibitor brefeldin A (BFA), leading to the intracellular accumulation of BFA bodies, and by the actin depolymerising agent latrunculin B (LatB; Geldner *et al*., 2001). To determine whether VAMP714 is also subject to actin‐dependent endosome recycling, we treated *proVAMP714::VAMP714:mCherry* seedlings with 50 μM BFA or 20 μM LatB, and monitored the formation of VAMP‐positive BFA bodies in root cells. We also treated *proPIN1::PIN1:GFP* and *proPIN2::PIN2:GFP* seedlings with 50 μM BFA as positive controls. The VAMP714:mCherry fusion protein was demonstrated to be biologically functional, as shown by transgenic complementation of the *vamp714* loss‐of‐function mutant (Fig. 1c). We found that VAMP714, PIN1 and PIN2 exhibit the same frequency of BFA body formation after 2 h treatment, which can be washed out (Fig. 8a; summarised in Fig. S8), indicative of endosome recycling between BFA compartments and the plasma membrane. Specifically, 48.4% of cells in *proPIN1::PIN1:GFP* roots (across all 20 plants analysed, 401 cells) showed BFA bodies; 54% in *proPIN2::PIN2:GFP* roots (all 20 plants, 447 cells); and 55.7% in *proVAMP714::VAMP714:mCherry* roots (all 20 plants, 447 cells). We also found that LatB caused intracellular accumulation of VAMP714 vesicles (Fig. 8b), with all seedlings analysed showing endomembrane compartment formation with an average incidence of 46.8% in root cells (20 plants, 481 cells). This suggests that VAMP714 forms part of both the exocytic vesicle‐trafficking pathway from the ER/Golgi and the actin‐dependent endocytic recycling pathway, which together regulate PIN protein concentrations at the plasma membrane. Relatively high intracellular levels of both PIN1:GFP and PIN2:GFP, and some intracellular PIN1:GFP‐positive vesicle‐like structures are seen in the *vamp714* mutant, dominant‐negative mutant and overexpressers compared with wild‐type (Figs 8c,S9), broadly consistent with the observations for PIN immunolocalisation (Fig. 7c) and indicative of a requirement of VAMP714 for polar PIN localisation.

We therefore also investigated the role of VAMP714 in endocytic recycling. We monitored PIN1 and PIN2 recycling in the *vamp714* loss‐of‐function (20 plants, total cell number 410 (PIN1) and 383 (PIN2)) and dominant‐negative mutants (20 plants, total cell number 410 (PIN1) and 390 (PIN2)) in the presence of 50 μM BFA. No PIN accumulation in BFA bodies was found to occur in either mutant background (20 plants, total cell number at least 400; Fig. 8c). This suggests that VAMP714 is required for PIN endosome recycling.

## Discussion

SNAREs have been classified as vesicle‐associated (v‐SNAREs) and target membrane‐associated SNAREs (t‐SNAREs) (Sollner *et al*., 1993), though under a structural classification they can be grouped as Q‐ and R‐SNAREs, owing to the occurrence of either a conserved glutamine or arginine residue in the centre of the SNARE domain (Fasshauer *et al*., 1998). Generally, t‐SNAREs correspond to Q‐SNAREs, and v‐SNAREs correspond to R‐SNAREs. There are more than 60 SNARE protein‐encoding genes represented in the Arabidopsis genome (Uemura *et* *al*., 2004; Lipka *et al*., 2007; Sanderfoot, 2007; Gu *et al*., 2020), but there is limited information available on the roles of SNARE proteins from genetic studies in plants, most likely because of a lack of loss‐of‐function phenotypes due to functional redundancy between related family members. For example, redundancy has been demonstrated between VTI11 and VTI12 (Kato *et al*., 2002; Surpin *et al*., 2003), SYP121 and SYP122 (Assaad *et al*., 2004; Zhang *et al*., 2008), and VAMP721 and VAMP722 (Kwon *et al*., 2008).

Some functional information on SNAREs in higher organisms is available from animal studies. The animal VAMP synaptobrevin has been implicated in linking synaptic vesicles to the plasma membrane (Walch‐Solimena *et al*., 1993; Bonifacino & Glick, 2004). It is proposed that R‐SNAREs may play a key role in determining specificity in vesicle budding, and an important mechanism for SNARE localisation is interaction with vesicle coats. For example, it has been shown that R‐SNAREs may be components of the COPII vesicles that are involved in ER–Golgi transport (Springer & Schekman, 1998), and that R‐SNAREs must be packaged into COPI vesicles during retrieval from the Golgi (Rein *et al*., 2002).

The data presented in this paper provide new information on both the role of the plant R‐SNARE VAMP714 in the control of auxin transport and auxin‐mediated responses via PIN protein expression, recycling and localisation. Directional (polar) auxin transport is key to establishing functional concentration gradients of auxin that mediate the control of cell identity, tropic growth and the nature of interactions with other hormones to elicit specific responses (Benjamins & Scheres, 2008). This process is mediated principally by PIN protein family members, some of which become localised to specific faces of the cell plasma membrane; and expression of *PIN* genes appears to reflect local auxin concentrations, reflective of a feedback regulatory system (Omelyanchuk *et al*., 2016). PIN localisation involves an actin‐mediated recycling between the plasma membrane and endosomes, providing a mechanism for rapid changes in the placement of these transporters (Geldner *et al*., 2001; Kleine‐Vehn *et al*., 2008).

We propose a model in which the correct delivery of PIN proteins from the ER/Golgi to the plasma membrane is via a VAMP714‐associated compartment, that is a necessary precursor to the endocytic recycling that provides dynamical control over the level and site of PIN protein localisation. This in turn regulates the direction and rate of auxin efflux. We show that VAMP714 is required for a range of correct auxin responses, including auxin‐mediated gene expression, root gravitropism, root cell patterning and shoot branching. The aberrant root meristem cell specification and organisation, and reduced root and hypocotyl lengths seen in VAMP714 loss‐of‐function and dominant‐negative mutants, and transgenic overexpressers, suggest a requirement for this gene in the control of cell division and expansion, and together with the observed defects in auxin gene expression, demonstrate a requirement of correct VAMP714 expression for correct auxin‐mediated responses. As seen in other systems, the phenotypic similarities between plants with loss‐of‐function and gain‐of‐function (over‐/misexpressers) of VAMP714 may be due to the disruption of interaction with partner proteins in both mutants and over‐/misexpressers (reviewed by Prelich, 2012), and this observation suggests that the stoichiometry of protein complexes in which VAMP714 is involved is required for correct function.

Our model is supported by the co‐localisation of VAMP714 and PIN proteins at the plasma membrane; the accumulation of PIN proteins in the cytoplasm in *vamp714* loss‐of‐function and dominant‐negative mutants and VAMP714 misexpressers; and the requirement for wild‐type levels of expression of *VAMP714* for BFA body formation (i.e. endosome recycling). We found that while both PIN1:GFP and VAMP714:CFP, and PIN2:GFP and VAMP714:CFP, co‐localise at the plasma membrane, this is less clear for PIN2 than for PIN1 (Fig. 7a,b). This may be linked to the stronger expression of the *VAMP714* gene in vascular tissues (Fig. S3), where PIN1 is also strongly expressed, while PIN2 is localised to epidermal and cortical cell layers where VAMP714 expression is less strong.

SNARES and Rab GTPases have been demonstrated to interact functionally to promote vesicle fusion at the endosome, and act coordinately to increase the specificity and efficiency of membrane fusion (Ohya *et al*., 2009; Ebine *et al*., 2011). We observe intracellular accumulation of PIN‐containing vesicles in the *vamp714* mutant, but future work is required to determine whether this represents a defect in exocytosis, endocytosis or both. Mechanistically VAMP714 may interact with the RAB5 GTPase complex known to participate in PIN recycling at the endosome (Dhonukshe *et al*., 2008), following its exocytic transport of PINs, and this possibility is the subject of further studies. It is also currently unclear whether VAMP714 is involved in transcytosis to modulate PIN localisation; and whether it is required for PIN‐specific or more general transport of plasma membrane proteins. These are areas for future research.

It is now well established that ARF‐GEF‐ and Rab5 GTPase‐dependent recycling is critical for PIN localisation, and this process is itself inhibited by BFA (an ARF‐GEF inhibitor) and modulated by auxin (Steinmann *et al*., 1999; Geldner *et al*., 2001; Kleine‐Vehn *et al*., 2008; Kitakura *et al*., 2011). Less clear have been the mechanisms regulating the exocytic delivery of PIN proteins from the ER/Golgi to the plasma membrane. We show in this paper that the Arabidopsis R‐SNARE VAMP714 is required for correct PIN localisation, likely via both the exocytic and endosome recycling pathways.

In the classical canalisation hypothesis pioneered by Sachs (Sachs, 1981), auxin itself promotes its own transport system, leading to directional flow through tissues and subsequent establishment of cell polarity and differentiation. Consistent with this hypothesis, the auxin‐mediated transcriptional activation of the *VAMP714* gene would allow the activation of a pathway essential for polar auxin transport by promoting correct PIN protein localisation at the plasma membrane. Integrated in this mechanism would be auxin‐mediated transcriptional effects on *PIN* genes (Heisler *et al*., 2005) and the effect of auxin on PIN endocytosis (Paciorek *et al*., 2005). A role for VAMP714 in the (probably indirect) maintenance of *PIN* gene expression is also indicated. Our studies demonstrate that R‐SNARE‐dependent exocytosis is essential for the auxin transport and downstream signalling pathways that are required for the control of cell polarity, tropic growth and morphogenesis in plants.

## Author contributions

KL, SAC and JFT devised the project; XG, KF, JA, AS and SAC carried out the experimental work; KL, JFT, PJH and GG supervised the work; KL drafted the manuscript; all authors edited the manuscript. XG and KF contributed equally to this work.

## Supporting information


**Fig. S1** Construction and analysis of dominant‐negative VAMP714 plants.
**Fig. S2** Root and hypocotyl lengths of mutants.
**Fig. S3** VAMP714 expression in Arabidopsis tissues.
**Fig. S4** VAMP7 family genes are auxin‐regulated.
**Fig. S5** Co‐localisation of PIN1:GFP and VAMP714:mCherry following transient expression in *Nicotiana benthamiana* leaf tissue.
**Fig. S6** PIN1 and PIN2 protein localisation in wild‐type and *vamp714* dominant‐negative mutant roots.
**Fig. S7** PIN3 and PIN4 protein localisation in wild‐type and *vamp714* dominant‐negative mutant roots.
**Fig. S8** Frequency of PIN1:GFP, PIN2:GFP and VAMP714:mCherry in endomembrane compartments following 50 μM BFA treatment for 2 h.
**Fig. S9** PIN1:GFP and PIN2:GFP distribution in cells of wild‐type, *vamp714* mutant and overexpressing seedling roots.

**Video S1** VAMP714 localises to the plasma membrane via vesicle trafficking.
Please note: Wiley Blackwell are not responsible for the content or functionality of any Supporting Information supplied by the authors. Any queries (other than missing material) should be directed to the *New Phytologist* Central Office.

## Data Availability

All materials and data described in this paper are available to readers from the corresponding author, upon reasonable request.
