# Supplementary material for: The Arabidopsis R‐SNARE VAMP714 is essential for polarisation of PIN proteins and auxin responses
Source: New Phytol. 2021 Feb 10;230(2):550–66. doi: 10.1111/nph.17205 (PMC8651015; doi:10.1111/nph.17205)
Supplement: Supplementary file 1 — Fig. S1 Construction and analysis of dominant‐negative VAMP714 plants. Fig. S2 Root and hypocotyl lengths of mutants. Fig. S3 VAMP714 expression in Arabidopsis tissues. Fig. S4 VAMP7 family genes are auxin‐regulated. Fig. S5 Co‐localisation of PIN1:GFP and VAMP714:mCherry following transient expression in Nicotiana benthamiana leaf tissue. Fig. S6 PIN1 and PIN2 protein localisation in wild‐type and vamp714 dominant‐negative mutant roots. Fig. S7 PIN3 and PIN4 protein localisation in wild‐type and vamp714 dominant‐negative mutant roots. Fig. S8 Frequency of PIN1:GFP, PIN2:GFP and VAMP714:mCherry in endomembrane compartments following 50 μM BFA treatment for 2 h. Fig. S9 PIN1:GFP and PIN2:GFP distribution in cells of wild‐type, vamp714 mutant and overexpressing seedling roots. [file NPH-230-550-s001.pdf]

New Phytologist Supporting Information

Article title: The Arabidopsis R-SNARE VAMP714 is essential for polarization of PIN proteins and auxin responses

Authors: Xiaoyan Gu, Kumari Fonseka, Julien Agneessens, Stuart A. Casson, Andrei Smertenko, Guangqin Guo, Jennifer F. Topping, Patrick J. Hussey and Keith Lindsey

Article acceptance date: 23 December 2020

Figure S1.

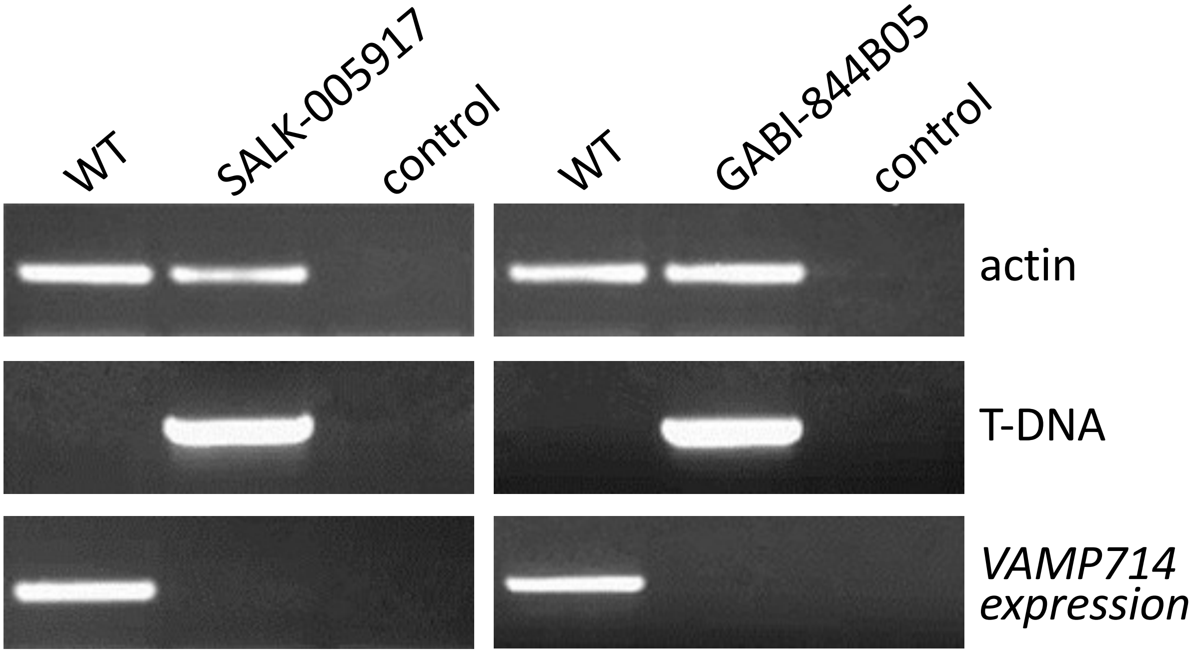

a. Genotyping SALK and GABI-KAT T-DNA insertion mutants. RT-PCR showing *ACTIN2* expression (upper panels), T-DNA presence (middle panels) and *VAMP714* expression (lower panels) in wildtype (WT), SALK-005917, GABI-844B05 seedlings and control samples lacking DNA/cDNA substrate, demonstrating the presence of T-DNA and loss of *VAMP714* expression in both T-DNA lines.

b. Domain structure of AtVAMP714.

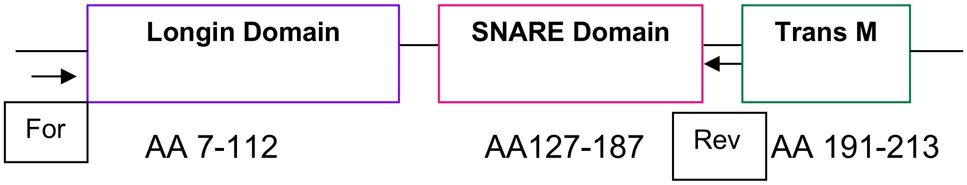

The three main domains of AtVAMP714 (Longin domain, SNARE domain and Transmembrane domain (Trans M) with the length of the amino acids. For and Rev indicates the position of forward and reverse primers designed for amplifying the Longin and SNARE domains, which were cloned into the Gateway vector pMDC43, under the transcriptional control of the CaMV35S gene promoter.

c. AtVAMP714 Domain DNA sequence and primers used.

For primer (Pink) and *Reverse* primer (Blue) were used to amplify the Longin and SNARE domains to create VAMP714 dominant negative transgenic plants.

```
At5g22360/ VAMP714
Longin Domain
SNARE Domain
Trans-membrane Domain
For primer
Reverse primer

   1  CATTTTTATA CTCTGTTCTG ATCGCAGCAA AGCCGACGTT GAACTTTCTC
  51  GCCGCCGGAG CGCGTGATCT CCACTCTCTG TCATCGAATC ACTCTAATTG
 101  AAGATTCTCC GATGGCGATT GTCTATGCTG TTGTAGCGAG AGGTACCGTG
 151  GTATTAGCTG AATTCAGCGC CGTTACGGGA AACACAGGCG CCGTGGTGCG
 201  ACGGATCCTC GAGAAGCTTT CACCGGAAAT CTCCGATGAA AGACTTTGTT
 251  TCTCTCAAGA TCGTTATATC TTCCATATTC TTAGATCTGA TGGTCTTACC
 301  TTTCTCTGTA TGGCCAATGA TACCTTTGGA AGGAGGGTTC CATTTTCGTA
 351  TTTGGAAGAG ATTCATATGA GATTCATGAA AAACTATGGC AAAGTGGCTC
 401  ATAATGCTCC AGCTTATGCA ATGAATGATG AATTCTCAAG GGTTTTGCAT
 451  CAGCAGATGG AGTTCTTCTC TAGTAATCCT AGTGTTGATA CTCTCAATCG
 501  TGTTAGAGGA GAAGTCAGTG AGATTCGATC GGTCATGGTA GAGAACATTG
 551  AGAAGATAAT GGAAAGAGGT GATAGGATTG AGCTTCTTGT TGATAAAACA
 601  GCAACAATGC AAGATAGCTC GTTTCACTTC AGGAAGCAAT CTAAGCGCCT
 651  TCGCCGAGCT CTTTGGATGA AAAATGCTAA GCTCCTGGTC TTGTTGACAT
 701  GCTTGATAGT TTTCTTGCTG TACATAATAA TCGCATCTTT CTGCGGAGGA
 751  ATCACTTTAC CATCATGCAG ATCTTAAAAT CTGGCGGCCT TATCTAAGGT
 801  ATACTGAAAC GGACCACTGT TTTTGTAAAT CAACTCAGTC GCATCATTTC
 851  GATTTGAAGC CTTGGTTTTG TCATGAAAAT GACTGTGAGT TTGAAGTTAC
 901  ATGTCATGGT CCTCCTTGCT TATGTAACTC TTGTAAATGT CAAAATCAAA
 951  ATGATACAGA GGTTCATTGA ACTCTTGCCT TGTCTTTTAG ATTTAGTCAC
1001  GTGAGTGAGT TTGTTCTCTG TATTTCCAAA ACTTTATCCG CTGTGTCTTT
1051  ATAATTTTTG CATTTGCAAT GTACATCACA TATTCGTTAT TTTTGTTGTT
1101  AAAATTACTT CAGTTTTCAT CTTTGTTTAA TAACATTTCT GATTCACAAA
1151  TA
```

d. **Domain amino acid sequences, highlighted in different colours (Longin, SNARE and Transmembrane).**

```
     Met Ala Ile Val Tyr Ala Val Val Ala Arg Gly Thr Val Val Leu Ala Glu    17
112  ATG GCG ATT GTC TAT GCT GTT GTA GCG AGA GGT ACC GTG GTA TTA GCT GAA

     Phe Ser Ala Val Thr Gly Asn Thr Gly Ala Val Val Arg Arg Ile Leu Glu    34
163  TTC AGC GCC GTT ACG GGA AAC ACA GGC GCC GTG GTG CGA CGG ATC CTC GAG

     Lys Leu Ser Pro Glu Ile Ser Asp Glu Arg Leu Cys Phe Ser Gln Asp Arg    51
214  AAG CTT TCA CCG GAA ATC TCC GAT GAA AGA CTT TGT TTC TCT CAA GAT CGT

     Tyr Ile Phe His Ile Leu Arg Ser Asp Gly Leu Thr Phe Leu Cys Met Ala    68
265  TAT ATC TTC CAT ATT CTT AGA TCT GAT GGT CTT ACC TTT CTC TGT ATG GCC

     Asn Asp Thr Phe Gly Arg Arg Val Pro Phe Ser Tyr Leu Glu Glu Ile His    85
316  AAT GAT ACC TTT GGA AGG AGG GTT CCA TTT TCG TAT TTG GAA GAG ATT CAT

     Met Arg Phe Met Lys Asn Tyr Gly Lys Val Ala His Asn Ala Pro Ala Tyr   102
367  ATG AGA TTC ATG AAA AAC TAT GGC AAA GTG GCT CAT AAT GCT CCA GCT TAT

     Ala Met Asn Asp Glu Phe Ser Arg Val Leu His Gln Gln Met Glu Phe Phe   119
418  GCA ATG AAT GAT GAA TTC TCA AGG GTT TTG CAT CAG CAG ATG GAG TTC TTC

     Ser Ser Asn Pro Ser Val Asp Thr Leu Asn Arg Val Arg Gly Glu Val Ser   136
469  TCT AGT AAT CCT AGT GTT GAT ACT CTC AAT CGT GTT AGA GGA GAA GTC AGT

     Glu Ile Arg Ser Val Met Val Glu Asn Ile Glu Lys Ile Met Glu Arg Gly   153
520  GAG ATT CGA TCG GTC ATG GTA GAG AAC ATT GAG AAG ATA ATG GAA AGA GGT

     Asp Arg Ile Glu Leu Leu Val Asp Lys Thr Ala Thr Met Gln Asp Ser Ser   170
571  GAT AGG ATT GAG CTT CTT GTT GAT AAA ACA GCA ACA ATG CAA GAT AGC TCG

     Phe His Phe Arg Lys Gln Ser Lys Arg Leu Arg Arg Ala Leu Trp Met Lys   187
622  TTT CAC TTC AGG AAG CAA TCT AAG CGC CTT CGC CGA GCT CTT TGG ATG AAA

     Asn Ala Lys Leu Leu Val Leu Leu Thr Cys Leu Ile Val Phe Leu Leu Tyr   204
673  AAT GCT AAG CTC CTG GTC TTG TTG ACA TGC TTG ATA GTT TTC TTG CTG TAC

     Ile Ile Ile Ala Ser Phe Cys Gly Gly Ile Thr Leu Pro Ser Cys Arg Ser   221
724  ATA ATA ATC GCA TCT TTC TGC GGA GGA ATC ACT TTA CCA TCA TGC AGA TCT
     Stop                                                                  221
```

e. *AtVAMP714* gene expression in dominant negative transgenics.

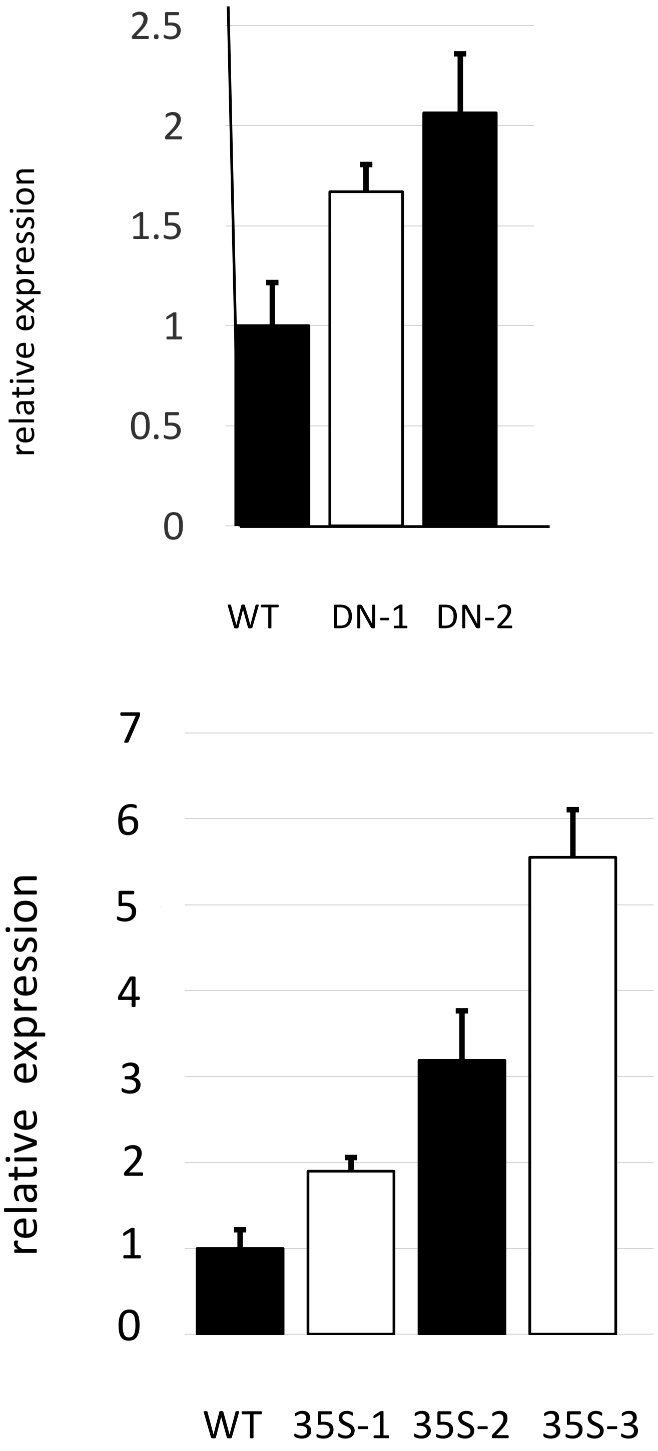

Quantitative RT-PCR analysis of *AtVAMP714* gene expression in two independent pro35S::VAMP714 dominant negative transgenic lines (upper panel) and three independent pro35S::VAMP714 lines (lower panel) compared with Col-0 wild type plants relative to *ACTIN2* at 7 days post germination**.** These data are representative of two independent experiments using biological replicate samples. The error bars represent

Standard Deviations of the mean of four technical replicates. Y axis represents the relative abundance of the *AtVAMP714* dominant negative (upper panel) or native (lower panel) transcript levels. PCR primers were designed to detect the truncated form of the dominant negative transcript (Longin and SNARE domains). The relative abundance of the respective *AtVAMP714* transcript was higher in transgenics than in Col-0 wild type plants.

f. Phenotypes of dominant negative and wildtype plants.

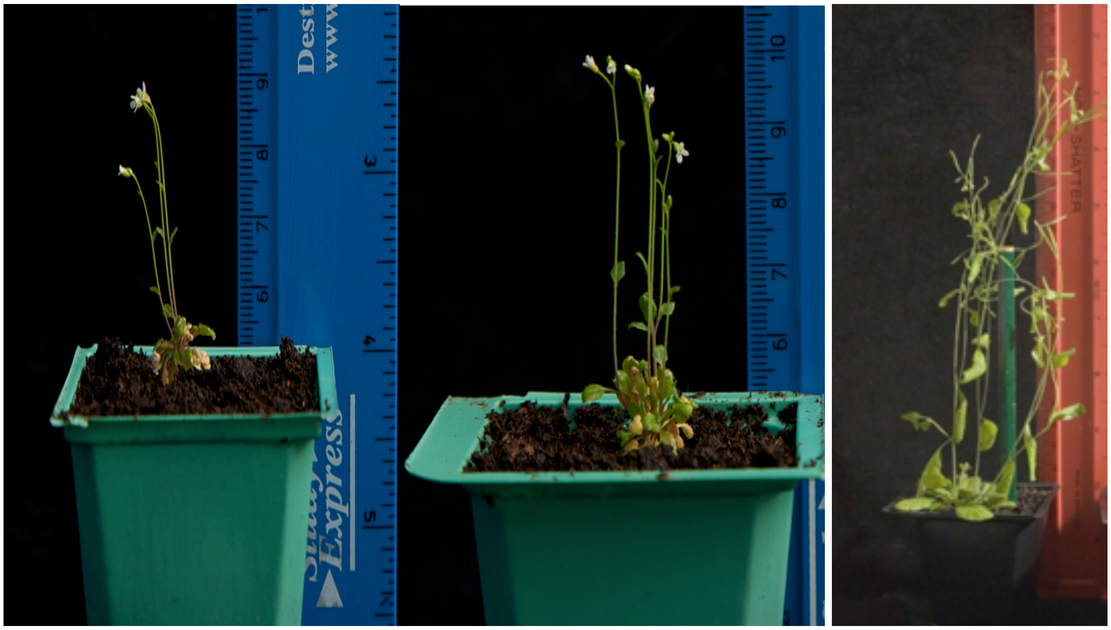

Left: Dominant negative transgenic plant at 21 dpg
Centre: Dominant negative transgenic plant at 40 dpg
Right: Col-0 wildtype plant at 40 dpg

g. Phenotypes of second dominant negative transgenic line showing similar phenotype (excess branching) at 21 dpg.

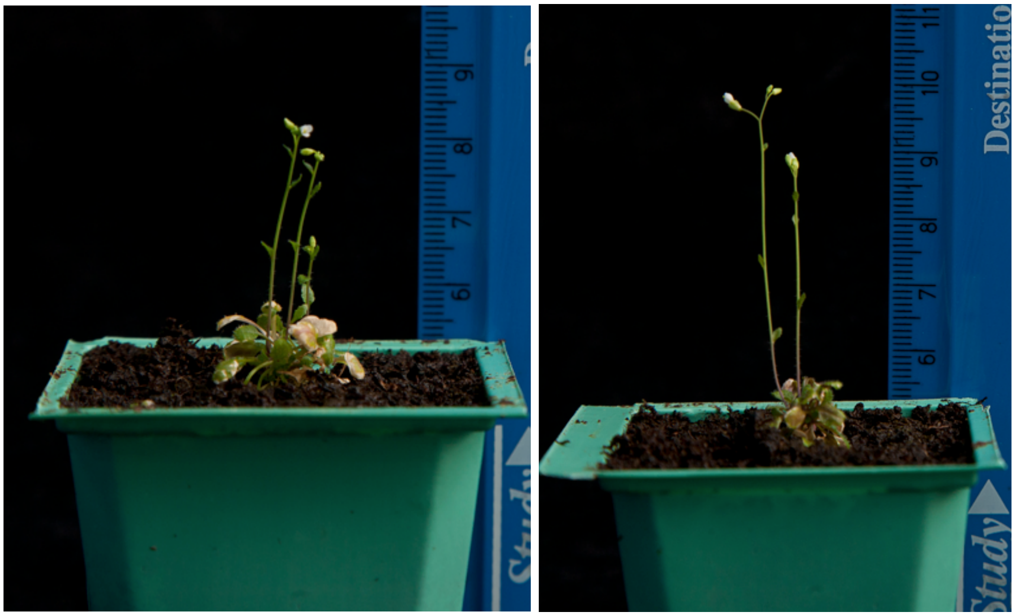

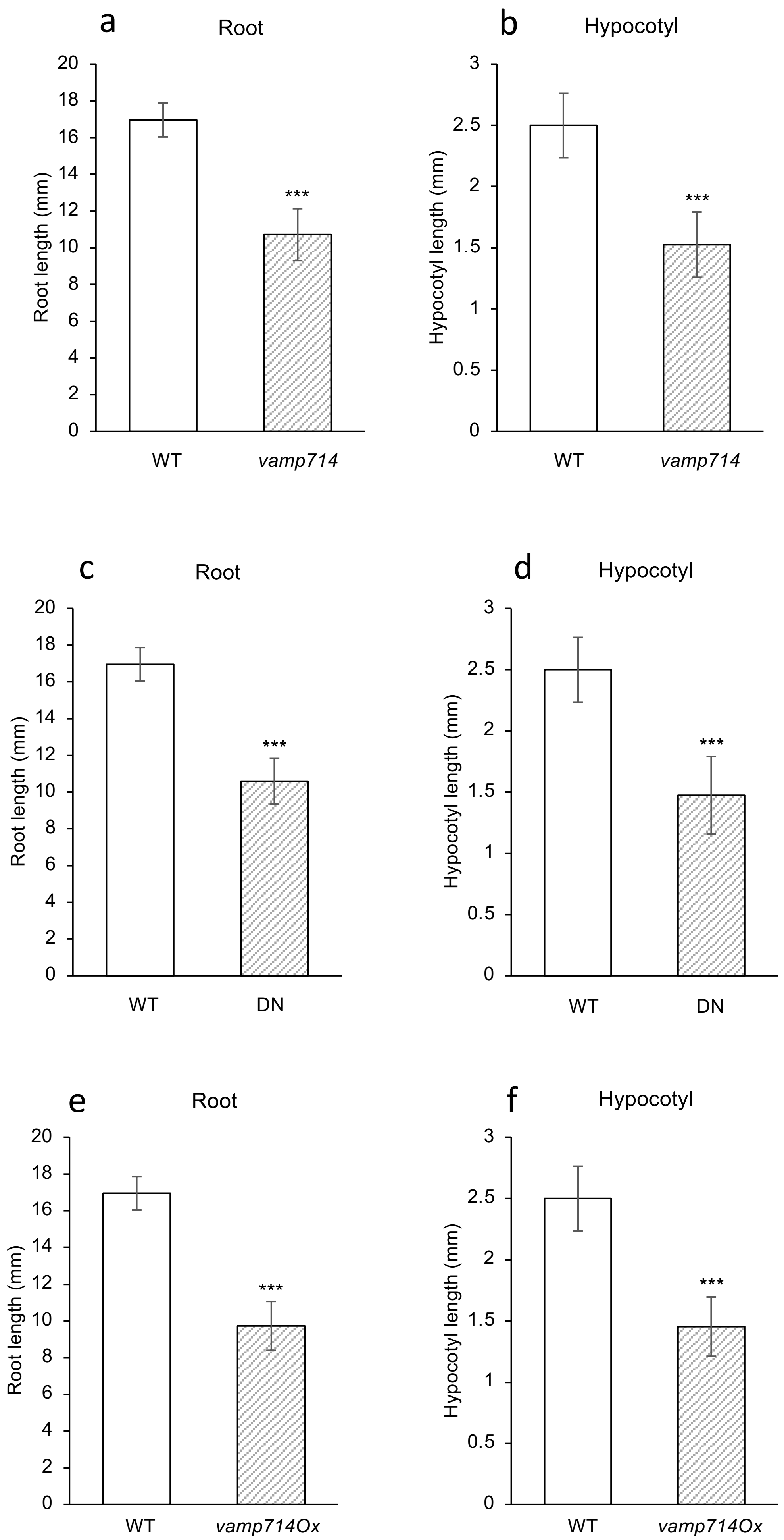
a
Root
Root length (mm)
20
18
16
14
12
10
8
6
4
2
0
***
WT
vamp714
b
Hypocotyl
Hypocotyl length (mm)
3
2.5
2
1.5
1
0.5
0
***
WT
vamp714
c
Root
Root length (mm)
***
WT
DN
d
Hypocotyl
Hypocotyl length (mm)
***
WT
DN
e
Root
Root length (mm)
***
WT
vamp714Ox
f
Hypocotyl
Hypocotyl length (mm)
***
WT
vamp714Ox

Fig. S2

Figure S2. Root and hypocotyl lengths of mutants.

Primary root and hypocotyl lengths of wildtype (WT) and *vamp714* loss-of-function mutants (a,b), dominant negative mutants (c,d) and transgenic VAMP714 overexpressers (e,f) grown on vertical agar plates for 7 dpg. Mean of 20 replicates ± standard error of the mean. *** shows significance at $P < 0.001$, Student's *t*-test.

Fig S3

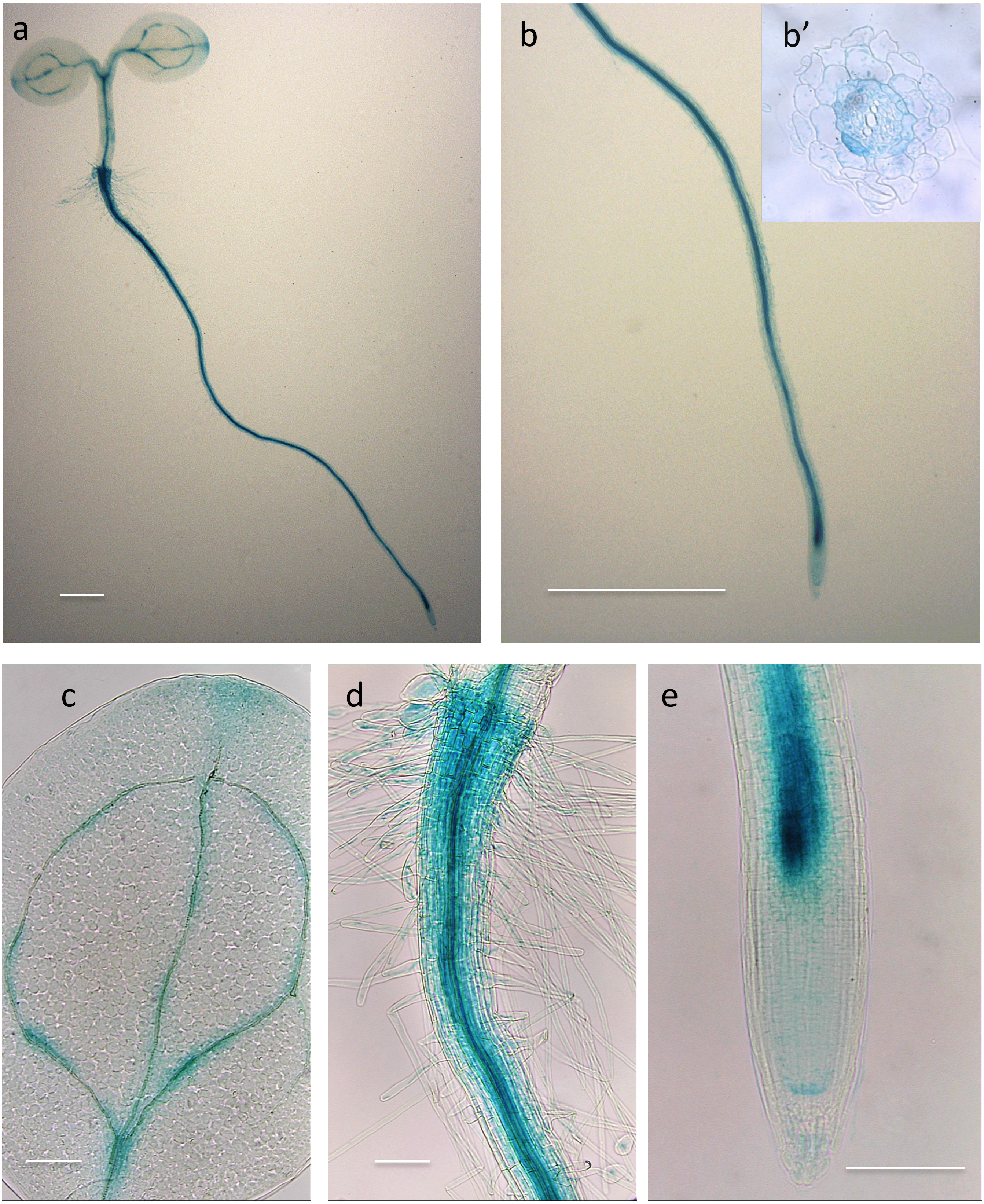

f

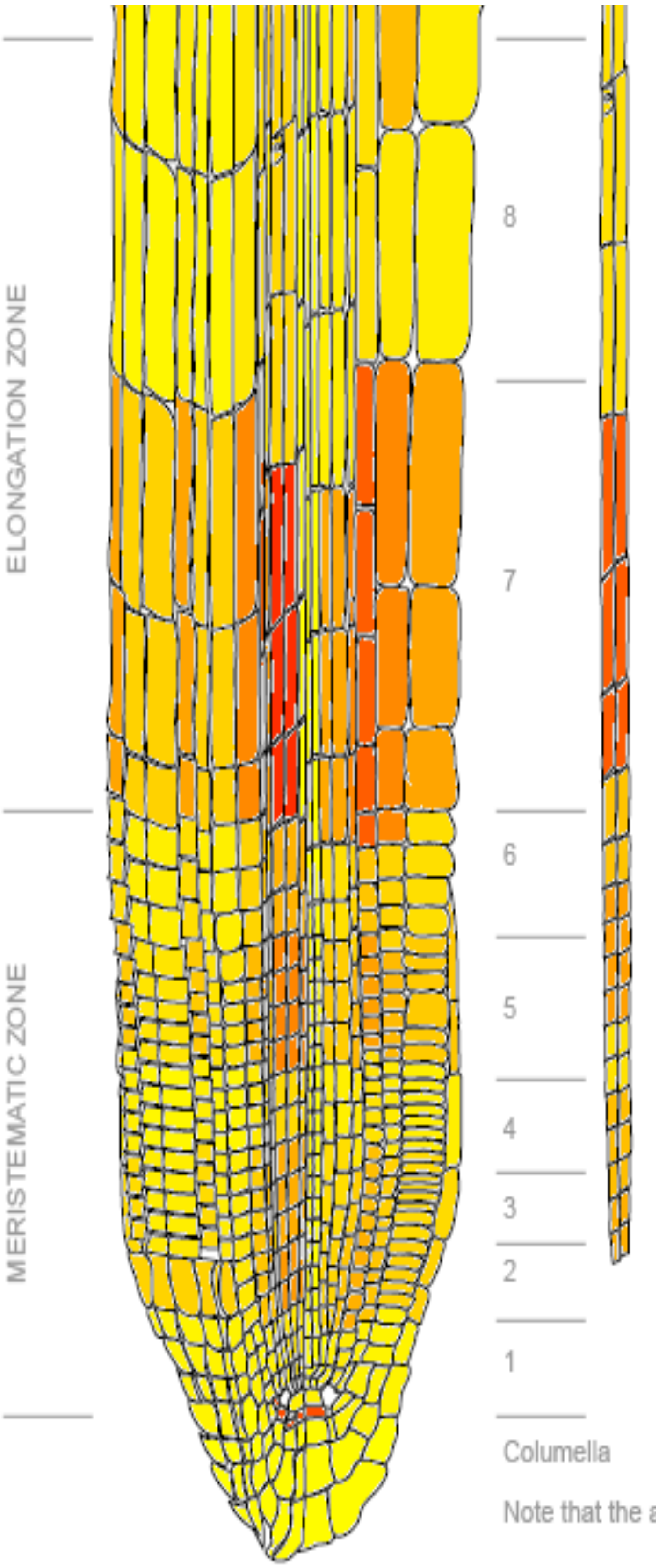

bar.utoronto.ca.

Figure S3. VAMP714 expression in Arabidopsis.
(a-e) *proVAMP714::GUS* is expressed in vascular tissues.
(a) Whole seedling, 4 dpg, bar = 1 mm.
(b) Seedling root, 4 dpg with transverse section in mature region of root, showing GUS activity in the stele (b'), bar = 1 mm.
(c) Cotyledon at 4 dpg, bar = 1 mm.
(d) root-hypocotyl junction at 4 dpg, bar = 1 mm.
(e) primary root tip, 4 dpg, bar = 100 µm.
(f) Expression heat map of VAMP714 gene in primary root of Arabidopsis. Visualized using online tool at http://bar.utoronto.ca/eplant/. Red denotes high expression, yellow denotes low expression.

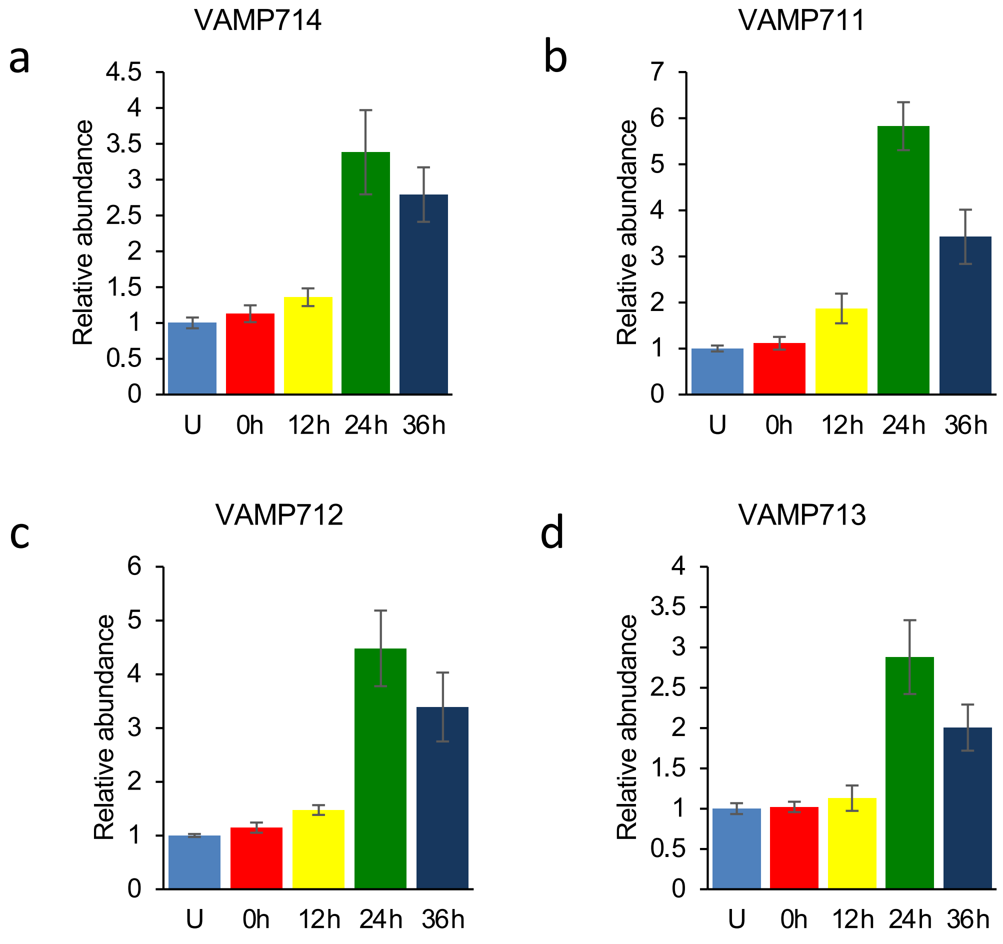

Figure S4. VAMP7 family genes are auxin-regulated.
qRT-PCR analysis of mRNA abundance of *VAMP7* family genes *VAMP714* (a), *VAMP711* (b), *VAMP712* (c) and *VAMP713* (d) in wildtype seedlings either untreated (U) or treated with 100 μM IAA for 0, 12, 24 and 36 h. Expression levels are relative to *ACTIN2* expression. Data represent means of 3 biological replicates ± SD.

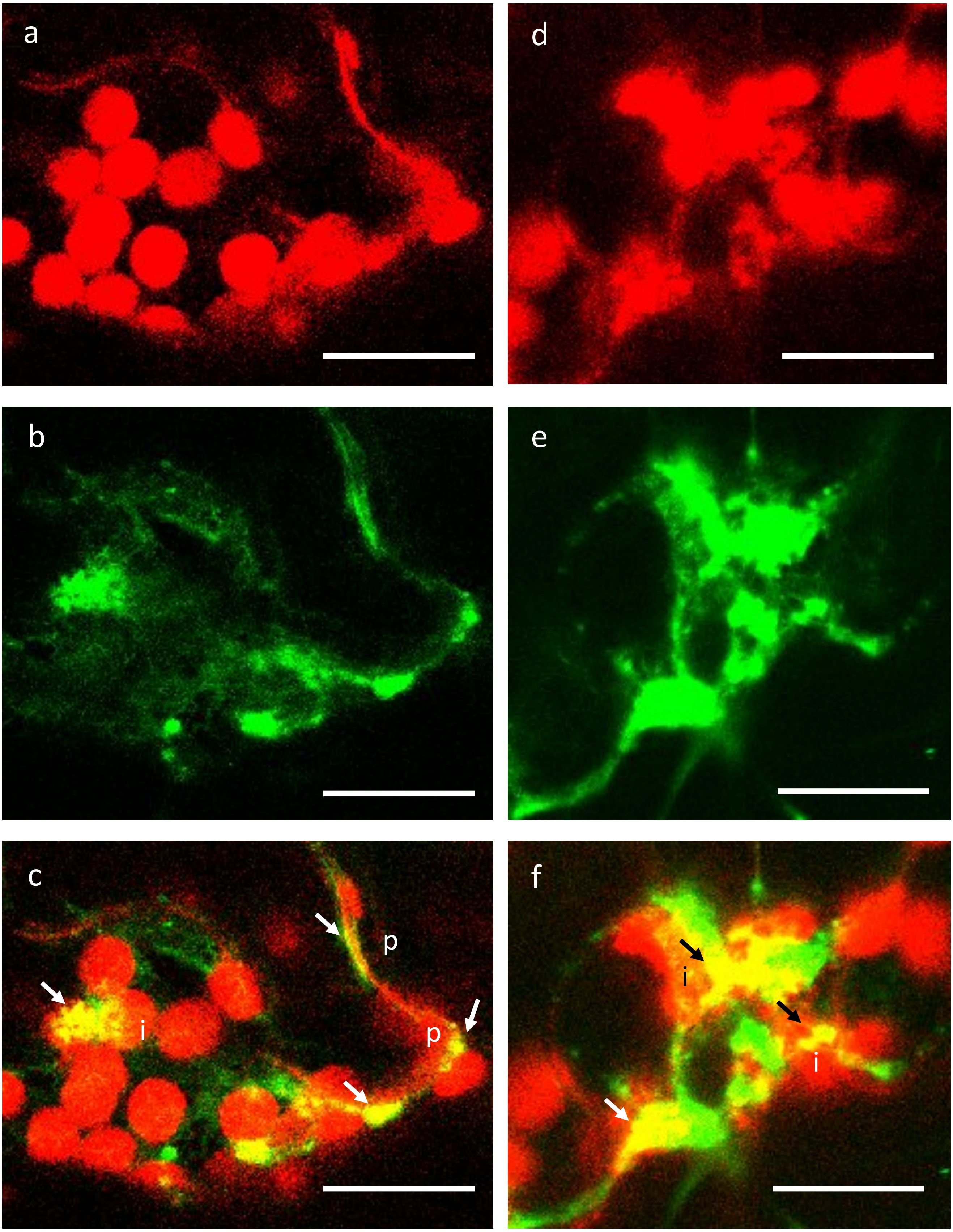

Fig. S5. Co-localization of PIN1:GFP and VAMP714:mCherry following transient expression in *Nicotiana benthamiana* leaf tissue. (a) VAMP714:mCherry localization; (b) PIN1:GFP localization; (c) images from (a) and (b) merged; (d) VAMP714:mCherry localization; (e) PIN1:GFP localization; (f) images from (d) and (e) merged. Arrows (both white and black) indicate merged signal in membranous structures at the cell periphery (p) and in internal membranous structures, possibly Golgi (i). Scale bars = 25 µm.

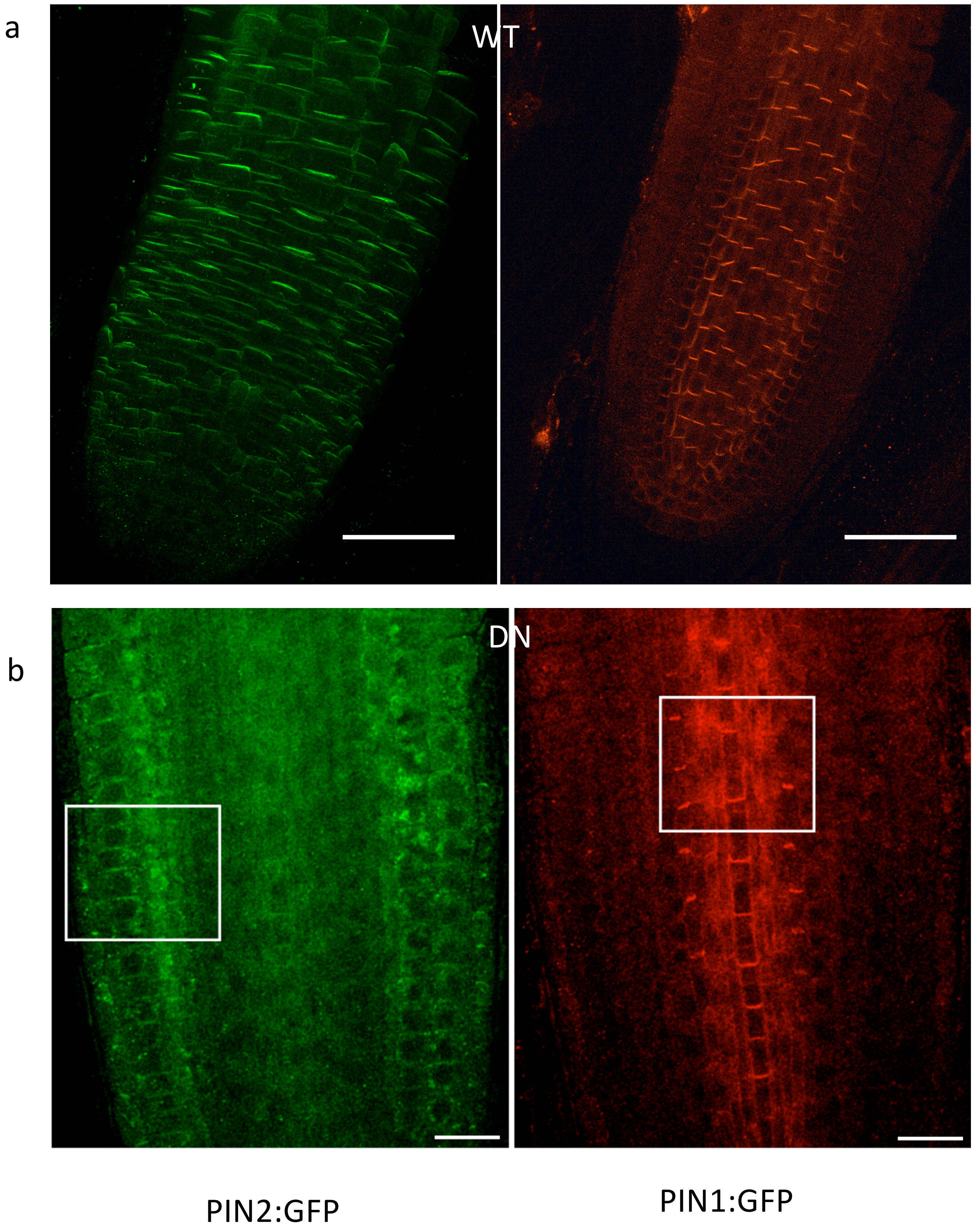

Figure S6. PIN1 and PIN2 protein localization in wildtype and *vamp714* dominant negative mutant roots. (a) PIN2 (left panel, green) and PIN1 (right panel, left) immunolocalization in wildtype (upper panels) and (b) *vamp714* dominant negative mutant (lower panels, PIN2: left; PIN1: right) roots. Boxes correspond to the central images in Fig. 7c. (a) bars = 25 µm; (b) bars = 20 µm.

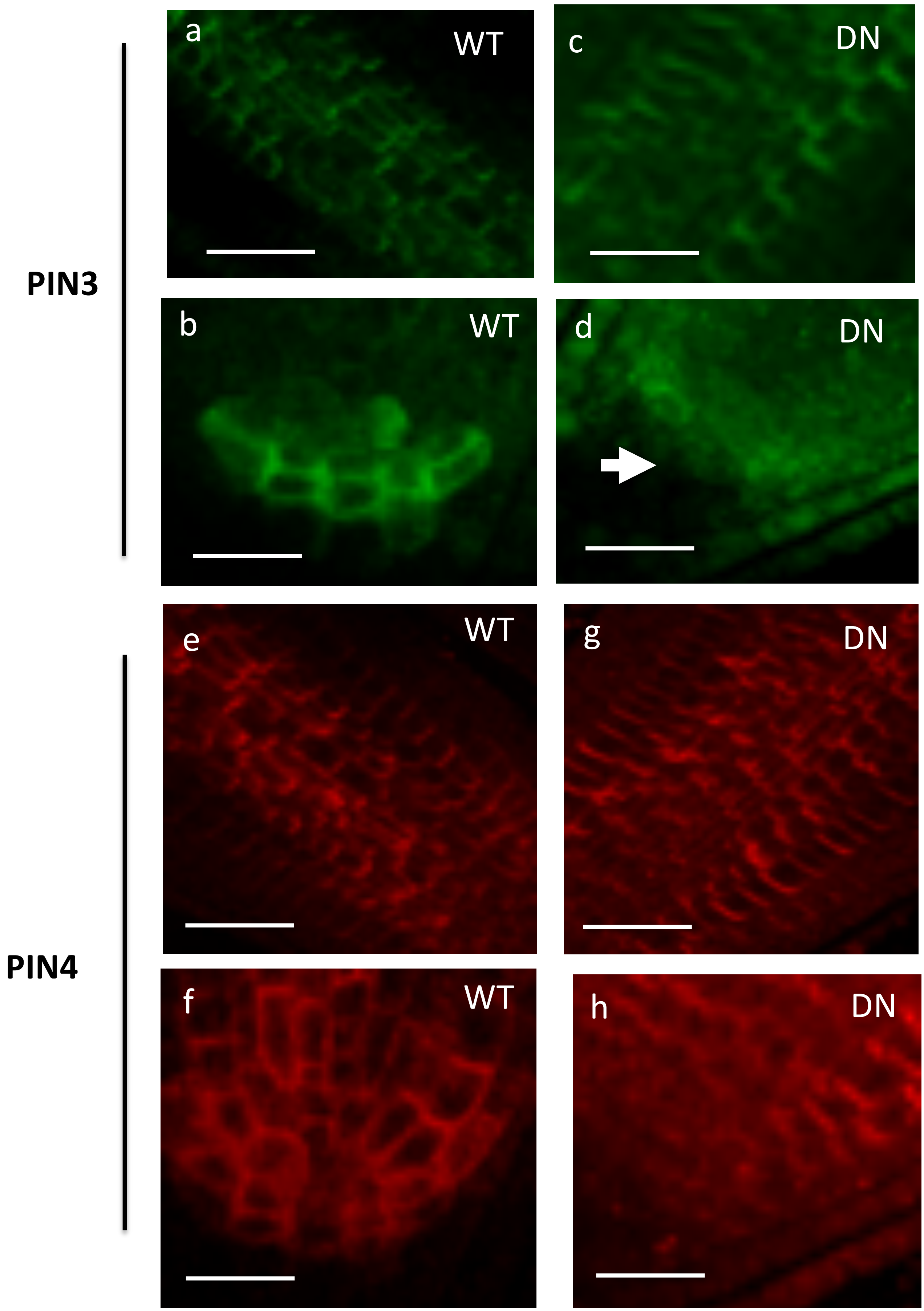

Fig. S5

Figure S7. PIN3 and PIN4 protein localization in wildtype and *vamp714* dominant negative mutant roots.

PIN3 (a-d) and PIN4 (e-h) immunolocalization in seedling roots of wildtype (WT, a,b,e,f) and *vamp714* dominant negative mutants (DN, c,d,g,h) at 7 dpg. (a,c,e,g) show cells in the proximal part of the meristem above the QC. (b,d) Cells in the position of the distal stem cell nice and first two tiers of. the columella, where PIN3 is strongly expressed in wildtype. The arrowhead in (d) shows complete lack of PIN3 at this position in the mutant. (f,g) Stem cell niche where PIN4 is most strongly expressed in wildtype. Expression in the mutant (h) is less distinct. (a,c,e,g) Bars = 25 µm. (b,d,f,g) Bars = 15 µm.

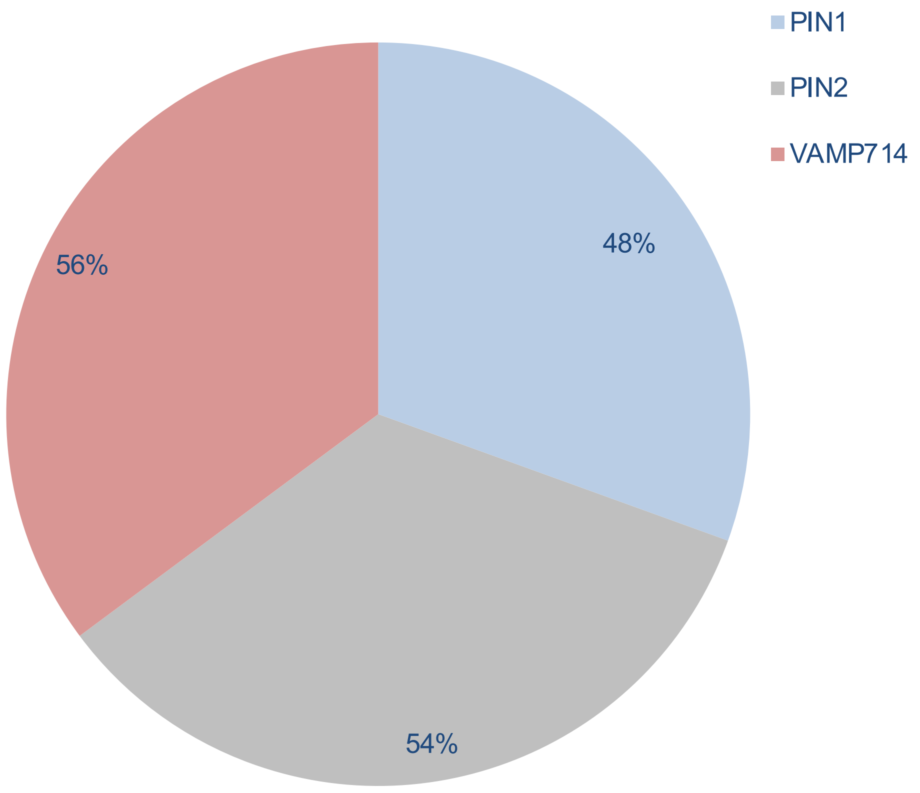

Fig. S8. Frequency of PIN1:GFP, PIN2:GFP and VAMP714mCherry in endomembrane compartments following 50 µM BFA treatment for 2 h. Endomembrane compartments were observed in all *proPIN1::PIN1:GFP* seedlings analysed with an average incidence of 48.4 % of root cells per plant (20 plants, total cell number = 401); in all *proPIN2::PIN2:GFP* seedlings analysed with an average incidence of 54.4 % of root cells per plant (20 plants, total cell number = 447); and in all *proVAMP714::VAMP714:mCherry* seedlings analysed with an average incidence of 55.7 % of root cells per plant (20 plants, total cell number = 488).

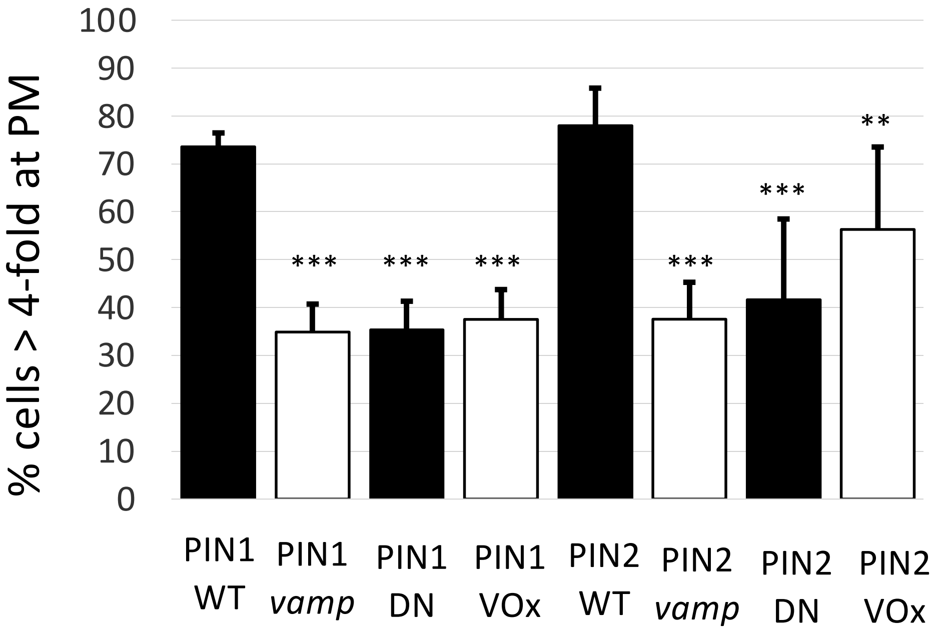

Figure S9. **PIN1:GFP and PIN2:GFP distribution in cells of wildtype, *vamp714* mutant and overexpressing seedling roots.** Quantification of PIN1:GFP and PIN2:GFP distribution in wildtype (WT), *vamp714* mutant *(vamp*), *vamp714* dominant negative (DN) and *VAMP714* overexpressing (VOx) seedling root cells, showing the percentage of cells with relatively strong fluorescence signal at the plasma membrane (four-fold above the cytoplasmic signal and above). Data are mean % ± SD of at least 30 cells per sample, 5 biological replicate samples. *** indicates significant difference between wildtype and mutants/overexpressers at $P < 0.005$, ** indicates $P < 0.05$, Student's *t*-test.

Video S1. VAMP714 localizes to the plasma membrane via vesicle trafficking. Video showing time series of VAMP714:mCherry expression (30 images were captured over 10 minutes).
